# Teratogenic Rubella Virus Alters the Endodermal Differentiation Capacity of Human Induced Pluripotent Stem Cells

**DOI:** 10.3390/cells8080870

**Published:** 2019-08-10

**Authors:** Nicole C. Bilz, Edith Willscher, Hans Binder, Janik Böhnke, Megan L. Stanifer, Denise Hübner, Steeve Boulant, Uwe G. Liebert, Claudia Claus

**Affiliations:** 1 Institute of Virology, University of Leipzig, 04103 Leipzig, Germany; 2 Interdisciplinary Center for Bioinformatics, University of Leipzig, 04107 Leipzig, Germany; 3 Schaller Research Group at CellNetworks, Department of Infectious Diseases, Virology, Heidelberg University Hospital, 69120 Heidelberg, Germany; 4 Research Group “Cellular Polarity and Viral Infection” (F140), German Cancer Research Center (DKFZ), 69120 Heidelberg, Germany

**Keywords:** ectoderm, mesoderm, human development, embryogenesis, interferon response, interferon-induced genes, self-organizing map (SOM) data portrayal, epigenetic signature, embryoid body, TGF-β and Wnt/β-catenin pathway

## Abstract

The study of congenital virus infections in humans requires suitable ex vivo platforms for the species-specific events during embryonal development. A prominent example for these infections is rubella virus (RV) which most commonly leads to defects in ear, heart, and eye development. We applied teratogenic RV to human induced pluripotent stem cells (iPSCs) followed by differentiation into cells of the three embryonic lineages (ecto-, meso-, and endoderm) as a cell culture model for blastocyst- and gastrulation-like stages. In the presence of RV, lineage-specific differentiation markers were expressed, indicating that lineage identity was maintained. However, portrait analysis of the transcriptomic expression signatures of all samples revealed that mock- and RV-infected endodermal cells were less related to each other than their ecto- and mesodermal counterparts. Markers for definitive endoderm were increased during RV infection. Profound alterations of the epigenetic landscape including the expression level of components of the chromatin remodeling complexes and an induction of type III interferons were found, especially after endodermal differentiation of RV-infected iPSCs. Moreover, the eye field transcription factors RAX and SIX3 and components of the gene set vasculogenesis were identified as dysregulated transcripts. Although iPSC morphology was maintained, the formation of embryoid bodies as three-dimensional cell aggregates and as such cellular adhesion capacity was impaired during RV infection. The correlation of the molecular alterations induced by RV during differentiation of iPSCs with the clinical signs of congenital rubella syndrome suggests mechanisms of viral impairment of human development.

## 1. Introduction

The enveloped, single stranded (positive-sense) RNA virus rubella virus (RV) of the genus *Rubivirus* within the family *Togaviridae* is one of the few viruses that can cause an intrauterine infection. How these viruses are transmitted vertically from the infected mother to the fetus and how they impact human development is only partially resolved. In the case of the very efficient teratogen RV, the human-specific symptoms are categorized as congenital rubella syndrome (CRS) with the classical triad of clinical symptoms being sensorineural deafness, congenital heart disease (including cardiovascular and vascular anomalies), and cataracts [1,2]. Heart defects in CRS may comprise ventricular/atrial septal defects, patent ductus arteriosus, and patent foramen ovale. In congenital rubella, ocular (ophthalmic) pathologies include cataract, microphthalmia, glaucoma, and pigmentary retinopathy [1,2]. Furthermore, in tissue samples from three fatal CRS cases RV was detected in cardiac and adventitia (aorta and pulmonary artery) fibroblasts in association with vascular lesions [3]. The risk for the development of congenital defects is especially prevalent during maternal rubella until gestational week 11 and 12 [4,5,6]. Thus, intrauterine RV infection is only of concern during the first trimester. While congenital malformations are common, premature delivery and stillbirths are not markedly increased after intrauterine RV infection [1].

There are a number of ethical constraints associated with the study of human embryogenesis and congenital malformations, especially as early implantation stages of human embryos are inaccessible [7]. With embryonic stem cells (ESCs) and induced pluripotent stem cells (iPSCs), as the two types of human pluripotent stem cells (PSCs), these novel ex vivo cell culture platforms allow for the analysis of human embryonic germ layer segregation and as well as for developmental toxicity testing [8]. As a cell culture model, they represent a blastocyst-like stage, which can be extended to gastrulation-like stages through their differentiation into derivatives of the embryonic germ layers (ectoderm, mesoderm and endoderm). Additionally, their suitability as a developmental model has been demonstrated for cardiac commitment during development [9] as the heart is the first organ to develop and cardiac cell fate decisions occur very early. Furthermore, cultivation of ESCs in combination with suitable 3D matrices or together with trophoblast cells enables the formation of blastoids, gastruloids, and even embryoids (or embryo-like entities) as culture dish models for human embryogenesis [7,10].

PSCs and PSC-based differentiation models, especially the mouse (m) ESC test, are already validated for testing of teratogenic and embryotoxic substances such as thalidomide (brand name Contergan^®^), [11,12]. However, their potential for the study of infections during pregnancy is just at the beginning of evaluation [13,14]. In line with the limited number of viruses that can cause perinatal infection, iPSCs possess intrinsic mechanisms that restrict virus infections. In addition, compared to differentiated somatic cells, iPSCs have a higher expression level of a distinct set of interferon (IFN)-induced genes [14]. This appears to counterbalance the absence of a type I IFN response in iPSCs as an essential component of antiviral innate immunity [15].

Teratogenic RV can be maintained in iPSCs over several passages followed by directed differentiation into embryonic germ layer cells [13], highlighting iPSCs as a promising model for the very early mechanisms involved in rubella embryopathy. As a follow-up to this study we aimed at the identification of RV-induced molecular alterations in these cells before and after initiation of directed differentiation through transcriptomics. The most profound effects associated with RV infection were detected in endodermal cells derived from RV-infected iPSCs. Markers for definitive endoderm were upregulated, which occurred in association with profound epigenetic changes, an upregulation of factors involved in vasculogenesis, and reduced activity of the TGF-β signaling pathway. Additionally, ectodermal cells revealed an altered expression profile of essential transcription factors for eye field development during RV infection. Thus, the study of RV infection on iPSCs and derived lineages provides insights into viral alterations of early developmental pathways and as such into congenital diseases in general.

## 2. Materials and Methods

### 2.1. Cell Lines and Cultivation

Vero (green monkey kidney epithelial cell line, ATCC CCL-81) and A549 (human lung carcinoma epithelial cells, ATCC, LGC Standards GmbH, Wesel, Germany) were cultured in Dulbecco’s modified Eagle’s medium (DMEM; Thermo Fisher Scientific, Darmstadt, Germany) with high glucose, GlutaMAX, 10% fetal calf serum (FCS) and 100 U/mL penicillin–streptomycin. If not otherwise indicated, the vector-free human episomal A18945 iPS cell line (alias TMOi001-A), (Thermo Fisher Scientific) was maintained in mTeSR™1 medium (StemCell Technologies, Cologne, Germany) with 10 µg/mL gentamycin on Matrigel™ (BD Biosciences, dispensed in DMEM/F-12)-coated culture plates with daily medium change. They were passaged enzymatically at a ratio of 1:6 to 1:10 every 3 to 5 days with collagenase type IV (Thermo Fisher Scientific) in DMEM-F12 with the addition of 10 μM Y-27632 ROCK inhibitor.

### 2.2. Directed and Undirected Differentiation of iPSCs

Directed differentiation was performed as an endpoint differentiation assay through the STEMdiff^TM^ trilineage differentiation kit (StemCell Technologies). The differentiation protocol was performed according to the manufacturer’s instructions and required cultivation of A18945 iPSCs in mTeSR™1 medium. Single cells, as obtained after treatment with Accutase (Merck/Sigma-Aldrich Chemie GmbH, Taufkirchen, Germany), were plated on Matrigel. Every 24 h medium change of the respective STEMdiff^TM^ trilineage differentiation medium for ectoderm, mesoderm, and endoderm was performed. Samples were collected after 5 days (mesoderm and endoderm) and 7 days (ectoderm) of cultivation. Undirected differentiation was initiated 24 h after collagenase-passaging of iPSC cultures at a ratio of 1:4 through application of undirected differentiation medium (DMEM-F12, 1x MEM-NEAA, 0.2 mM L-glutamine, 20% FBS, 0.11 mM β-mercaptoethanol, and 100 U/mL penicillin) followed by further cultivation for 5 days.

### 2.3. Embryoid Body Formation

EB formation as based on a previous publication [16] and (http://www.biolamina.com/media.ashx/instructions-bl010.pdf) was carried out in suspension culture and single cell suspensions were obtained after Accutase (Sigma-Aldrich) treatment. A total of 1 × 10^6^ cells was seeded in 200 µL of EB culture medium (DMEM-F12, 20% KnockOut™ Serum Replacement (Thermo Fisher Scientific), 1× MEM-NEAA, 0.2 mM L-glutamine, 0.11 mM β-mercaptoethanol and 1 mg/mL Gentamicin) medium into one well of a nontreated conical 96-well plate and centrifuged at 600× *g* for 5 min. After cultivation for 2 days the EBs were transferred according to the protocol to a low attachment flat-bottom six-well plate and medium was changed every third day.

### 2.4. Virus Infection and Interferon Assays

The supernatant of infected Vero cells was collected and cleared from cellular debris by centrifugation at 350× *g* for 10 min at 4 °C and filtration through a 0.45 µm syringe filter. Thereafter ultracentrifugation with a 20% sucrose cushion (*w*/*v* in PBS) was performed for 2 h at 25,000 rpm and 4 °C. The obtained pellets were resuspended in mTeSR1. Viral titers were determined by standard plaque assay. As described previously [13], iPSC cultures with a 40–50% confluency were acutely infected with 7.5 × 10^5^ plaque forming units (PFU) of RV per well of a 24-well plate. This corresponds approximately to an MOI of 20. The applied MOI can only be estimated as iPSCs were passaged enzymatically in clumps. The inoculum was replaced with fresh mTesR1 medium after 2 h of incubation [13]. After 4 to 5 days of cultivation, RV-infected iPSCs were passaged.

For exogenous (or paracrine) IFN treatment human recombinant IFN lambda 1 (IL-29, #300-02L) and 2 (IL28A, #300-02K), were purchased from Peprotech (Hamburg, Germany), and 3 (IL-28B, #CS26) from Novoprotein (Novoprotein, PELOBIOTECH GmbH, Planegg/Martinsried, Germany). The Accuri C6 flow cytometer (BD Bioscience, Heidelberg, Germany) was used for IFN measurement by the LEGENDplex human type 1/2/3 IFN panel (BioLegend, San Diego, CA, USA). The double-stranded (ds) RNA analogue polyinosinic-polycytidylic acid (poly I:C; Santa Cruz Biotechnology, Heidelberg, Germany) was added either directly to the cell culture or transfected at a concentration of 1 µg using Lipofectamine 2000 (Thermo Fisher Scientific) as transfection reagent.

### 2.5. Calcein Live Cell Staining

For live cell staining, EBs were incubated with mTeSR1 plus calcein FM (Sigma-Aldrich) at 1 µM. After an incubation period for 30 min, EBs were washed twice with PBS and analyzed on an inverted fluorescence microscope.

### 2.6. RNA Isolation

Total RNA was extracted from mock- and RV-infected cells by Trizol reagent (Thermo Fisher Scientific). The purification was performed with the Direct-zol RNA kit (Zymo Research, Freiburg, Germany) according to manufacturer’s instructions. The integrity of the RNA samples was confirmed through analysis on a fragment analyzer (Advanced Analytical). Only samples with a RIN (RNA integrity number as a means of quality assessment) equal to 7 or greater were subjected to further analysis.

### 2.7. Microarray Gene Expression Analysis and SOM Portrayal

Isolated RNA was processed and hybridized to Illumina HT-12 v4 Expression BeadChips (Illumina, San Diego, CA, USA) and measured on the Illumina HiScan. Raw intensity data of 47,323 gene probes was extracted by Illumina GenomeStudio and subsequently background corrected, transformed into log_10_-scale, quantile normalized, and centralized to obtain gene expression estimates. Two independent samples per condition and cell type were processed.

Expression data were then further processed using self-organizing map (SOM) machine learning. The method distributes the gene-centered expression values among 2500 microclusters called meta-genes, which were arranged in a two-dimensional 50 × 50 lattice and colored in maroon-to-blue for high-to-low meta-gene expression values. These mosaic images visualize the transcriptome patterns of each individual sample and therefore can be understood as their molecular portraits exhibiting clusters of coexpressed genes in the samples studied [17]. Mean portraits over replicates were calculated by averaging the meta-gene landscapes of replicated samples while difference portraits between different cell types were obtained by subtracting the respective metagene values to highlight differentially expressed genes. Clusters of coexpressed genes were identified by selecting so-called ‘spot-areas’ in the SOM portraits using overexpression criteria as described previously [17]. For functional interpretation of the expression-modules, we applied gene set enrichment analysis using the gene set Z-score (GSZ), [17]. Enrichment of functional gene sets in the spot cluster was calculated by applying Fisher’s exact test. We considered gene sets related to biological processes (BP) of the gene ontology (GO) classification, standard literature sets [17,18], and literature sets curated by our group. Downstream analysis methods were described previously [17,19] and are implemented in the R-package ‘oposSOM’ used for analysis [20].

Pathway activity was analyzed based on pathway topologies and gene expression data using the pathway signal flow method as implemented in oposSOM [21].

### 2.8. Quantitative Real-Time PCR Analysis of Viral and Cellular RNA

For determination of the mRNA expression level of selected cellular genes, 1.2 µg of total RNA were reverse transcribed with Oligo(dT)_18_ primer and AMV reverse transcriptase (Promega, Mannheim, Germany) at 42 °C for 1 h. This was followed by an incubation step at 70 °C for 10 min. The carousel-based LightCycler 2.0 (Roche, Mannheim, Germany) was used for quantitative real-time PCR (qRT-PCR) experiments. These experiments included a 1:5 dilution of the respective cDNA samples together with 1 µg BSA and the *GoTaq^®^ qPCR master mix* (Promega). Supplement Table S1 lists oligonucleotides and probes targeting viral p90 gene that were used for quantification of viral RNA as described [22]. Two different approaches for relative expression analysis were pursued. For direct comparison of one sample type after mock- and RV-infection, comparative delta delta Ct (∆∆Ct) was used. For comparison of gene expression levels among different cell types within a large data set, a modified version of the comparative delta delta Ct (∆∆Ct) method was used. The normalized relative quantity (NRQ) values were derived from qbase+ software (Biogazelle, Zulte, Belgium) which are based on the mean expression values of all samples and replicates within a given data set [23].

### 2.9. Immunofluorescence

For assessment of viral proteins, immunofluorescence was carried out as described [13]. Briefly, cells were fixed with 2% (*w*/*v*) paraformaldehyde in PBS and permeabilized with 0.1 Triton X-100 followed by incubation with mAb anti-E1 from Viral Antigens (Viral Antigens Incorporation, Memphis, TN, USA) at a 1:200 dilution as primary antibody.

### 2.10. Statistical Analysis

All statistical calculations were done with Graph Pad Prism software (GraphPad Software, Inc., La Jolla, CA, USA). Asterisks (* *p* < 0.05, ** *p* < 0.01, *** *p* < 0.001, **** *p* < 0.0001) highlight the level of significance in diagrams which include data as means ± standard deviation (SD). For comparison of normalized mRNA expression levels in RV-infected samples with the corresponding mock controls, a paired Student’s *t* test (consistent ratios of paired values) was applied. Statistical analysis for different samples was based on one-way ANOVA followed by Bonferroni’s multiple comparison test.

## 3. Results

### 3.1. In the Presence of RV, iPSCs Maintain Pluripotent Properties and Lineage Identity after Initiation of Differentiation

**Figure 1:**
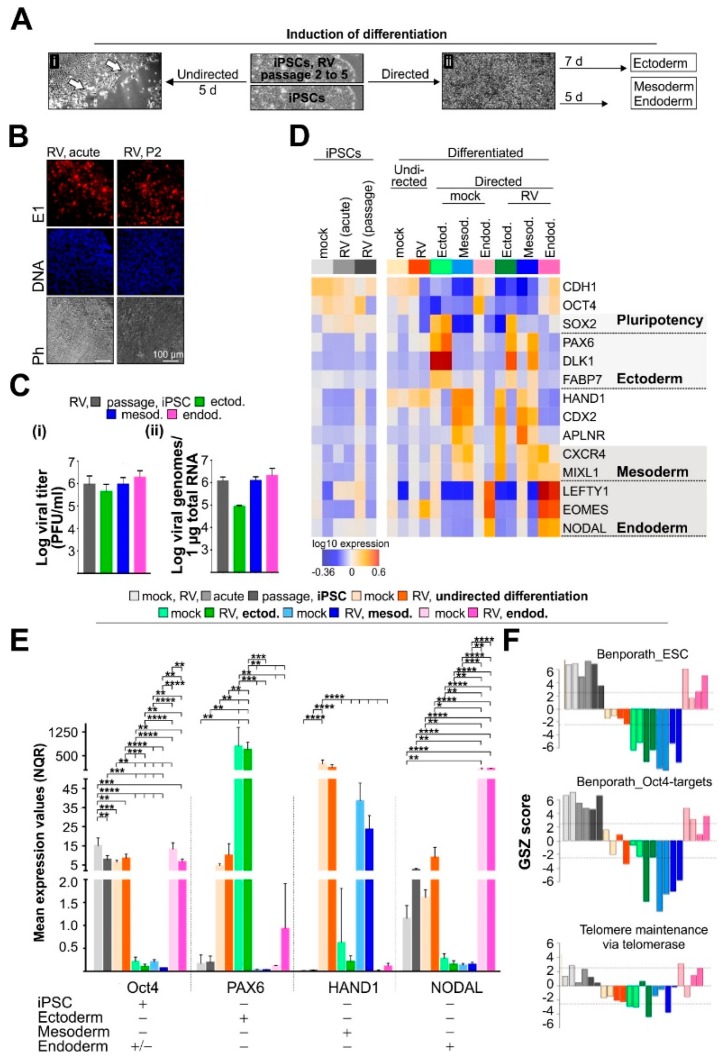
The identity of iPSCs and derived lineages was maintained in the presence of rubella virus (RV). (**A**) Overview of the methods applied to initiate differentiation in mock- and RV-infected iPSCs. (**A**) [**i**] Undirected differentiation in the respective induction medium started at the rim region of iPSC colonies (indicated by white arrows) and extended to their center over time of incubation. (**A**) [**ii**] Additionally, directed differentiation into the primary germ layers ectoderm, mesoderm, and endoderm was induced with the STEMdiff differentiation kit. (**B**) Immunofluorescence analysis with anti-E1 antibody (shown in red) was performed to monitor distribution of RV-positive cells within iPSC colonies. Nuclei are shown in blue. Ph, phase contrast (**C**) To assess RV replication in iPSCs and derived lineages, (**i**) virus progeny, and (**ii**) the amount of genomic viral RNAs was determined by standard plaque assay (n = 11 for passaged iPSCs, otherwise n = 3) and TaqMan-based reverse transcription-quantitative PCR (passaged iPSCs and ectodermal cells n = 3, mesodermal cells n = 7, endodermal cells n = 4), respectively. (**D**) Expression heatmap of selected marker genes of pluripotency and lineage identity (based on microarray whole transcriptome data) in mock- and RV-infected iPSCs and iPSC-derived lineages. Shading indicates overlap in the expression of some of the marker genes between iPSCs and iPSC-derived lineage cells, respectively. (**E**) The expression of indicated target genes in mock- and Wb-12-infected iPSCs and derived cells after initiation of undirected and directed differentiation was determined by real-time quantitative PCR (RT-qPCR) and analyzed by qbase+ software. For normalization, chromosome 1 open reading frame 43 (C1orf43) and hypoxanthine guanine phosphoribosyl transferase 1 (HPRT1) were used. Relative gene expression was calculated as normalized relative quantity (NRQ) and given as means ± SD (n = 3 to 5). As a reference, lineage-specific expression levels were assigned based on literature data and transcriptomics data by Stemcell Technologies for the Trilineage differentiation kit. (**F**) Analysis of stemness-related expression signatures was based on the GO gene set telomere maintenance. An embryonal stem cell signature and a gene set collecting OCT4 targets were both taken from [25].

Specification to one of the three germ lineages is the first critical step in directing differentiation to downstream cellular phenotypes. Therefore, directed as well as undirected differentiation was induced in RV-infected iPSCs which were subcultured for two to five passages. Passaging of infected iPSCs results in a homogenous level of infection within iPSC cultures without affecting the protein expression level of the pluripotency marker OCT4 [13]. During passaging of RV-infected iPSCs, replication occurred at a rather constant rate as assessed by viral titer and E1 protein expression rate [13]. Furthermore, passaging allows for adaptation of RV to iPSCs and excludes any possible effects of the differentiation process itself on the otherwise acute infection with RV (Figure 1A). Undirected differentiation is spontaneous and was thus induced to assess whether RV, without a specific differentiation stimulus, directs a gene expression profile different from the mock-infected population. Directed differentiation of RV-infected iPSCs into ecto-, endo-, or mesodermal cells (thereafter referred to as RV-infected) was initiated with the STEMdiff trilineage differentiation kit as an endpoint differentiation approach to determine which of the early cell fate decision pathways could be affected by RV.

RV establishes a noncytopathic infection of iPSCs with a homogenous distribution of infected cells within the respective colony (Figure 1B), [13]. Differentiation into all three embryonic germ layers supported RV replication at a comparable rate (Figure 1C [i,ii]), [13]. As a next step, we generated an expression heatmap of selected marker genes (based on microarray whole transcriptome data) for assessment of pluripotency and lineage identity (Figure 1D). In agreement with the maintenance of OCT4 (octamer-binding transcription factor 4, also known as POU5F1) expression in RV-infected iPSCs [13], high expression of pluripotency markers CDH1 and OCT4 was noted. Their expression was maintained to some degree in endodermal cells, which is in agreement with the conditions of the STEMdiff trilineage differentiation kit. The same applies to the expression of the pluripotency marker SOX2 (SRY (Sex Determining Region Y)-Box 2) in ectodermal cells. The expression profile of lineage-specific markers confirmed ectodermal (PAX6 (Paired Box 6), DLK1 (Delta-Like 1 Homolog), and FABP7 (Fatty Acid Binding Protein 7)), mesodermal (HAND1 (Heart and Neural Crest-Derived Transcript 1), CDX2 (Caudal Type Homeobox 2), APLNR (Apelin Receptor)) and endodermal (LEFTY1 (Left-Right Determination Factor 1), EOMES (Eomesodermin), NODAL (Nodal Growth Differentiation Factor)) identity after initiation of directed differentiation in mock- and RV-infected iPSCs (Figure 1D). Additionally, some overlap between lineages, especially between mesoderm and endoderm, was noted for RV-infected samples (Figure 1D). Transcriptomic data was confirmed by RT-qPCR of pluripotency marker OCT4 and of selected markers for ectoderm (PAX6), mesoderm (HAND1), and endoderm (NODAL) lineages followed by relative quantification by qbase+ method (Figure 1E). Figure 1D and E indicate that during undirected differentiation, especially mesodermal markers were expressed, which occurred at a comparable level between mock- and RV-infected cells. Among the lineage-specific markers, the expression level of HAND1 was significantly downregulated in mesodermal cells after RV infection. Additionally, RV infection did not alter stemness-related expression signatures as indicated by the transcriptomic activity of the GO gene set telomere maintenance (Figure 1F). Telomere maintenance is active in stem cells, but gets deactivated in differentiated somatic cells [24].

In summary, comparable to the mock-control, RV maintained the pluripotent properties of iPSCs and enabled initiation of differentiation into embryonic germ layer cells as indicated by expression of essential germ layer markers.

### 3.2. High-Resolution Transcriptomic Maps Reveal Modules of Coregulated Genes Promoted by RV Infection during Endodermal Differentiation

**Figure 2:**
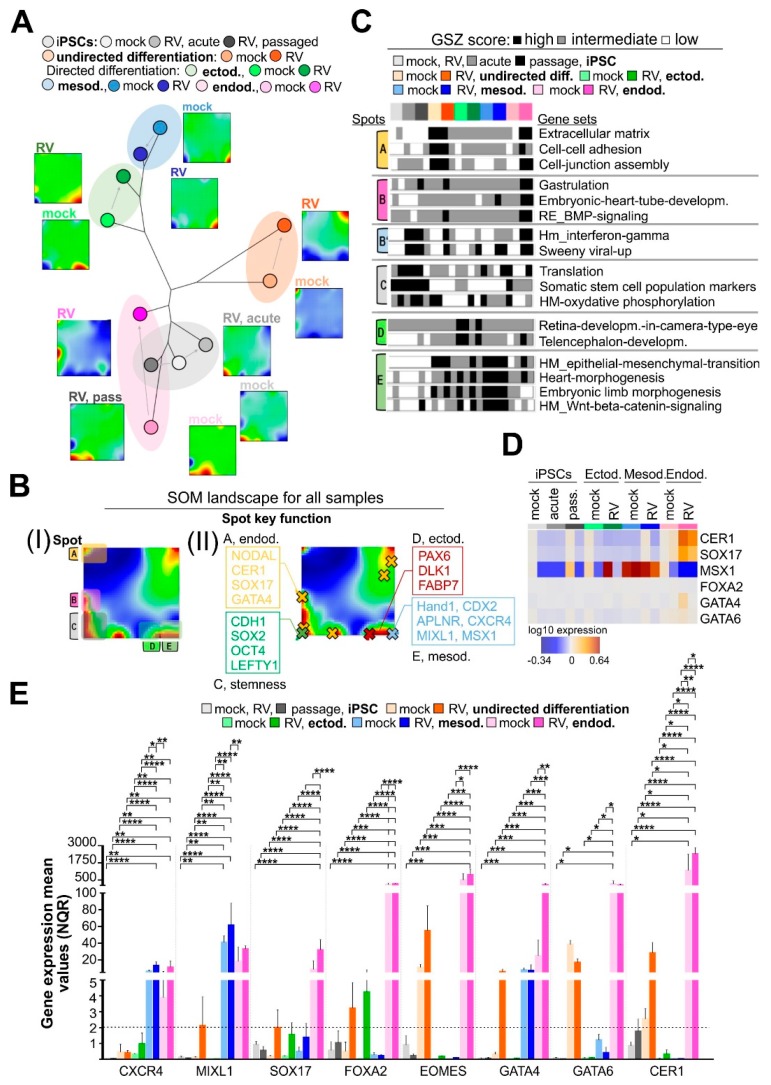
RV supports expression of markers for definite endoderm. (**A**) Similarity tree of the gene expression portraits of the cell systems studied. Both, mock- and RV-infected mesodermal and ectodermal cells share relatively high mutual similarity of their transcriptomes. In contrast, mock- and RV-infected endodermal cells form a separate branch that is closer to the mock- and RV-infected iPSCs. (**B**) Spot summary map (**I**) provides an overview of activated cellular programs and their functional context, which is depicted in more detail in (**II**). (**C**) Barplot representation of the expression profiles of gene sets related to different functions. Their genes were enriched in the spots that were identified in the SOM portraits. They are thus indicated accordingly (see also the overview map in part B of the Figure and Table S2). (**D**) Selected marker genes for definite endoderm are illustrated in the heatmap of the transcriptome of mock- and RV-infected iPSCs and iPSC-derived lineages. (**E**) The expression of marker genes for definite endoderm was determined by RT-qPCR in mock- and Wb-12-infected iPSCs and iPSC-derived lineages by qbase+ software. For normalization, C1orf43 and HPRT1 were used. Relative gene expression was calculated as normalized relative quantity (NRQ) and given as means ± SD (n = 3 to 5). See also Figures S1 and S2.

As we found out that RV infection did not affect unspecific differentiation of iPSCs and enabled their lineage-specific differentiation, we wanted to focus on the effect of RV on lineage identity. The self-organizing map (SOM) transcriptome data portrayal provides a high-resolution visualization of the transcriptome landscape of each cell system studied in terms of a quadratic mosaic image and decomposes into clusters of coregulated genes. They are represented as colored spot-like areas where red and blue colors code activated and deactivated gene clusters, respectively. These transcriptomic portraits were then used to evaluate the mutual relatedness between the cell systems by means of a phylogenetic similarity tree (Figure 2A). The tree structure results from the fact that common and different spot patterns in the portraits reflect mutual similarities and differences of the activated cellular programs which enable judging the effect of RV-infection on the different lineages (see the portraits in Figure 2A). For an overview, we generated a spot-summary map in Figure 2B which shows the activated spots observed in any of the samples together with their functional context as extracted by means of gene set enrichment analysis of the genes in each of the spot-clusters of coexpressed genes (see also Supplement Table S1). In total, we identified five relevant spots labeled with capital letters A–E. Each of the spots is characterized by a specific expression profile (Supplement Figures S1 and S2) which, in turn, shows close similarities with the expression profiles of distinct gene sets (shown as ‘barcode’ plots in Figure 2C).

Mapping of the position of genes into the SOM enables us to deduce their expression profile in the cell systems studied according to the gene’s location in or near the spots and the respective spot profiles (Figure 2B and Supplement Figure S1). Importantly, key genes of ectoderm and mesoderm development are found in spots D and E, respectively, and are confirmed to be upregulated in the ectoderm and mesoderm cells. On the other hand, genes related to gastrulation and heart-tube development were enriched in spot B and found to be activated in RV-infected endoderm cells. This occurred together with genes that are activated upon interferon response (see next subsection). Stemness key genes locate in spot C together with part of the developmental genes of the endoderm (Supplement Figure S1). Difference portraits clearly indicate that spot B associates with RV infection (Figure 2B), which contains the genes NODAL, CER1, SOX17, and GATA4, reflecting their activation upon RV infection in endodermal cells. In addition, we are able to show that mesoderm and ectoderm cells share similar expression patterns characterized by upregulated spots D and E and downregulated stemness genes (spot C), which, in contrast, are upregulated in endoderm cells. This is in agreement with the expression level of pluripotency genes in the endodermal lineage which was higher than in the remaining two embryonal germ layers (Figure 1D,E). As a consequence, mesoderm and ectoderm cells occupy neighboring positions at one end of the similarity tree, while endoderm cells are found at the opposite end. These findings highlight two important aspects: (I) there is a close similarity of transcriptional patterns among ecto-, mesoderm, and iPSC samples without and with RV infection; and (II) the endodermal lineage is an exception, where RV infection induces a notable shift away from the corresponding mock sample.

To further elucidate this specific effect of RV, we analyzed the transcriptomic data for markers for definitive endoderm (CXCR4 (C-X-C Motif Chemokine Receptor 4), MIXL1, SOX17, FOXA2 (Forkhead Box A2), EOMES, GATA6, CER1 (Cerberus, DAN family BMP Antagonist), and LEFTY, [26]. The expression heatmap shown in Figure 2D indicates an increased expression level of CER1 and SOX17 after RV infection. Figure 2E shows qPCR analysis followed by relative quantification by qbase+ method and highlights that the definite endoderm markers CXCR4, MIXL1, SOX17, GATA4/6, and CER1 were significantly higher expressed in RV-infected endodermal cells as compared to their mock-infected counterparts. While RV infection did not induce these markers above a cut-off of a two-fold increase during mesodermal and ectodermal differentiation, EOMES, GATA4, and CER1 were specifically induced by RV during undirected differentiation.

In conclusion, similarity analysis supports the hypothesis that ectodermal and mesodermal lineage identity was maintained after infection with RV, while endodermal cells derived from RV-infected iPSCs were enriched in markers for definitive endoderm.

### 3.3. RV Infection Activates IFN Type III Response Pathways on iPSCs and Derived Lineages

**Figure 3:**
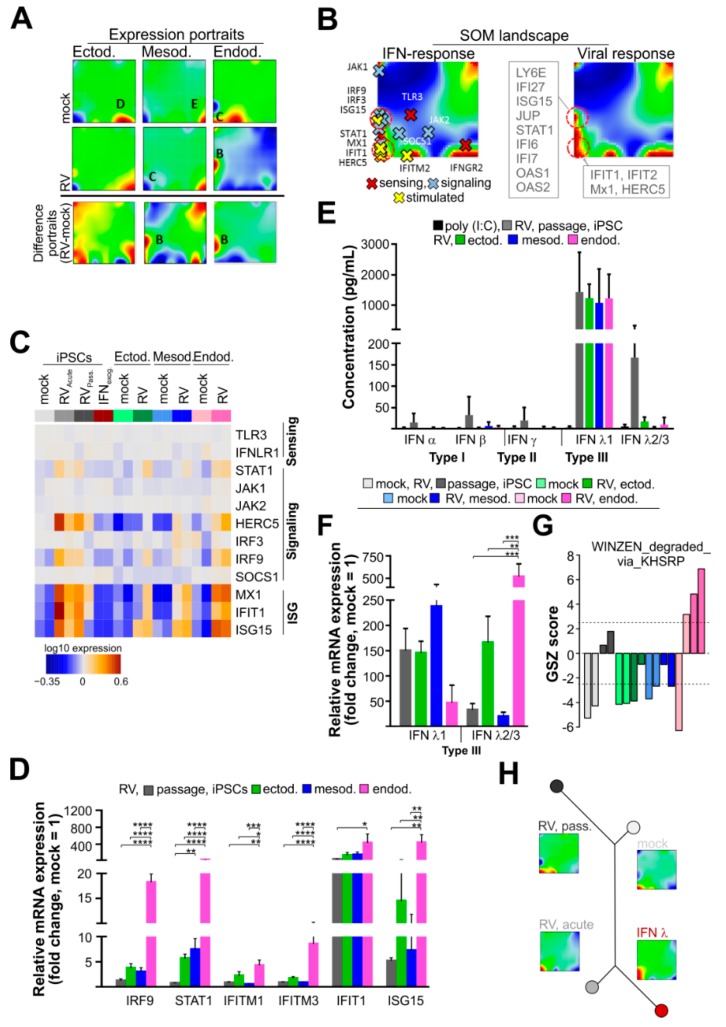
RV infection activates an IFN response in iPSCs and derived lineages. (**A**) Expression portraits of the embryonal germ layers before and after RV infection and the respective difference portraits reveal characteristic spot patterns, where spots C, D, and E are specific for endoderm, ectoderm, and mesoderm cells, respectively. Spot B appears after RV infection mainly in endodermal cells. (**B**) IFN response genes with signaling and stimulated functions in the IFN response pathways accumulate in spots B and C and were upregulated predominantly in iPS and endodermal cells after RV infection as also indicated by the IFN and viral response gene signature profiles. (**C**) Expression heatmap of selected marker genes involved in IFN-sensing and -signaling in mock- and RV-infected iPSCs and iPSC-derived lineages. Interferon-stimulated genes (ISGs) that were identified by the SOM analysis shown in (**A**) are included. (**D**) The mRNA expression level of the IFN-signaling components IRF9 and STAT1 and indicated ISGs was verified by RT-qPCR analysis. Data are given as means ± SD (n = 3 and n = 5 for RV-infected mesoderm, IRF9 and STAT1). (**E**) The IFN profile for RV-infected iPSCs and derived lineages was determined by the LEGENDplex IFN panel for undiluted supernatants collected after five (iPSCs and mesodermal cells) and seven days (endodermal and ectodermal) of cultivation. (**F**) The mRNA expression level of type III IFNs was verified by qPCR analysis and given as means ± SD (n = 3). (**G**) Mean expression of a gene set (gene set Z-score, GSZ) that is controlled by KSRP, which appears to keep inflammatory gene expression within defined limits [31]. (**H**) Similarity tree of the gene expression portraits of mock- and RV-infected iPSCs in comparison to iPSCs after cultivation in the presence of exogenous type III IFNs. (**D**,**F**) For normalization of qRT-PCR data in the 2^−∆∆Ct^ method, the HPRT1 gene was used. See also Figure S3.

Difference portraits (Figure 3A) indicated that RV-infection specifically upregulated genes in spot B, which were associated with “IFN response” characteristics. This is supported by the profiles of gene expression signatures of viral infections such as by influenza virus and pneumonia that is accompanied by interferon activation. These genes were consistently upregulated in RV-infected samples with the largest observed effect in endodermal cells (Supplement Figure S3), [27,28,29]. Spot B highlighted in the SOM landscapes contained genes involved in IFN and viral response mechanisms (Figure 3A, a list of genes is given in Figure 3B). This is further emphasized by the expression heatmap shown in Figure 3C. Whereas genes involved in IFN-sensing, including the type III IFN receptor IFNLR1 (IFN lambda receptor 1), were not altered in their expression level, the IFN-signaling components STAT1 (signal transducer and activator of transcription 1) and IRF9 (interferon regulatory factor 9) appeared to be slightly upregulated at their mRNA expression level after infection with RV, especially in endodermal cells. The highest level of upregulation was found for IFN-stimulated genes (ISGs), notably for MX1 (MX dynamin like GTPase 1), IFITM2 (interferon induced transmembrane protein 2), and ISG15 (interferon-stimulated gene 15). Mapping of these genes into SOM space further underlines these findings: The IFN-signaling genes and the ISGs accumulate in and around spots B and D, respectively, while IFN-sensing genes are located outside these spot regions (Figure 3B). The increase in the expression level of selected marker genes of the IFN pathway in the presence of RV was confirmed by RT-qPCR (Figure 3D). Compared to RV-infected iPSCs and ecto- and mesodermal cells, the expression level of IRF9 and STAT1 and selected ISGs (IFITM1/2, IFIT1, and ISG15) was significantly higher in RV-infected endodermal cells. The highest increase in mRNA expression after RV infection was noted for the ISGs IFIT1 and ISG15. Therefore, we determined whether this gene expression pattern was indeed associated with IFN generation during RV infection through quantification of type I (α and β), type II (γ), and type III (λ1 and λ2/3) IFNs by the LEGENDplex assay from the supernatants of RV-infected cells (Figure 3E). In iPSCs as well as iPSC-derived lineage cells, RV infection induced secretion of type III IFNs, namely IFN λ2/3 (Figure 3E). As a positive control, the synthetic dsRNA analog poly I:C was used, which was either transfected into iPSCs or added directly to the supernatant. Either application of poly I:C did not lead to secretion of any type I, II, or III interferons (Figure 3E). The activation of type III IFNs by RV was also confirmed at the mRNA level by RT-qPCR (Figure 3F). Compared to the mock-infected control, RV induced a significant increase in the mRNA expression of IFN λ2/3 in endodermal cells (Figure 3F). Thereafter, we addressed the discrepancy between IFN λ2/3 protein (Figure 3E) and mRNA level (Figure 3F). Gene set analysis revealed that mRNAs associated with the KH type-splicing regulatory protein (KHSRP) were specifically enriched in endodermal cells (Figure 3G). KHSRP is involved in post-transcriptional regulation of mRNA expression, including IFN λ3 [30]. This could explain the discrepancy between IFN λ2/3 protein and mRNA expression level.

To address the influence of the type III IFNs secreted during RV to the cell culture supernatant on the gene expression landscape of iPSCs, type III IFNs were added exogenously for two weeks of cultivation during daily medium change of iPSCs. The zoom-in similarity tree shown in Figure 3H highlights the relatedness between the expression portraits of mock- and RV-infected iPSCs as well as iPSCs after application of type III IFNs. The gene expression profile of passaged RV-infected cells shifted away from iPSCs after exogenous IFN type III application, but closer to the mock-infected cells, suggesting an adaptation of RV to iPSCs.

In conclusion, in iPSCs and iPSC-derived embryonic lineages, RV infection induced a type III IFN response together with activation of ISGs, notably MX1 and ISG15. This activation appeared to be specifically profound in endodermal cells.

### 3.4. RV Infection Is Associated with Chromatin Remodeling

**Figure 4:**
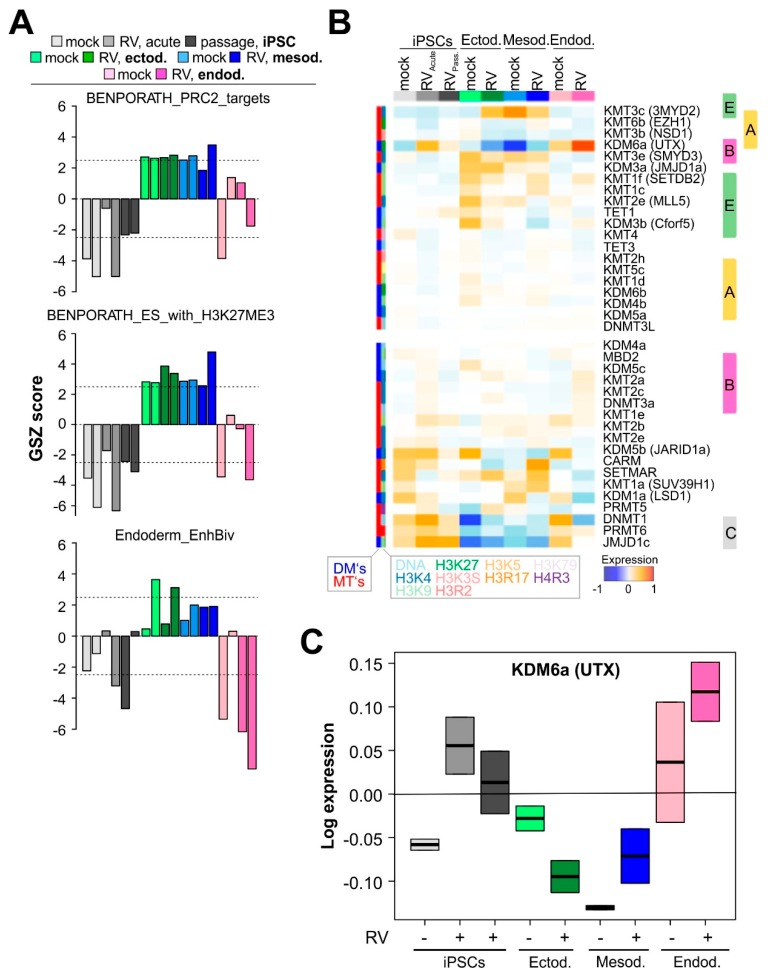
RV infection is accompanied by an altered expression of components of the SWI/SNF and NURF chromatin remodeling complexes. (**A**) Mean GSZ expression signatures of stemness-related transcriptional programs which act via epigenetic programming (PRC2 targets, repressive and bivalent chromatin marks). (**B**) Expression heatmap of chromatin modifying enzymes in the cell systems studied. The gene expression data of methyltransferases (MTs) and demethylases (DMs) of DNA cytosines, histone lysine and arginine side chains were assigned to the expression spot-cluster A–E according to their expression profiles. This suggests their involvement in the regulation of chromatin structure as writers and erasers of methylation marks at histone lysine and arginine side chains and affecting DNA methylation. Notably, a very strong variability was observed for KDM6a (alias UTX), a constituent of the SWI/SNF ATP-dependent chromatin remodeling machinery. (**C**) Expression of KDM6a directly relates to stemness programs (Figure 1F) and inversely relates to programs repressing stemness functions (part A of the Figure). See also Figure S4.

Alterations of gene expression patterns during development are governed by epigenetic mechanisms in cooperation with regulation via transcription factor networks [32,33]. Particularly, we found that gene signatures of epigenetic impact, such as targets of the polycomb repressive complex 2 (PRC2), of H3K27me3, and of bivalently (H3K4me3 and H3K27me3) marked gene promoters, have an almost antagonistic expression profile as compared to the stemness signatures (Figure 4A, in comparison to Figure 1F).

Next, we focused on chromatin remodeling which is essential for lineage segregation [9,34]. The analysis of transcription factor networks that act in regions of euchromatin with transcriptionally active genes and regulatory elements revealed that genes in repressed and bivalent states of endoderm progenitors become more rigorously deactivated in RV-infected endoderm cells than genes from these states of mesoderm progenitors in RV-infected mesoderm cells (Supplement Figure S4A). This suggests that RV infection specifically dedifferentiates endoderm cells by suppressing developmental suppressors. We then studied enzymes affecting methylation of DNA and of arginine and lysine side chains of histones such as H3K4, H3K9, H3K27, and H3K36, with potential impact on chromatin structure. The profiles of the DNA methylation maintenance methyltransferase DNMT1, of PRMT6, a methyltransferase of histone arginine side chains, and of JMJD1c, a H3K9 demethylase, and KDM5b demethylating H3K4me3 correlate with the ‘stemness’ spot cluster C upregulated in iPS and endodermal cells.

Notably, the gene encoding the H3K4 demethylase KDM6a (alias UTX) was markedly upregulated in RV-infected iPSCs and, especially, endoderm-derived cells (Figure 4B). KDM6a is a constituent of the SWI/SNF ATP-dependent chromatin remodeling machinery. Figure 4C highlights that in comparison to iPSCs and ecto- and mesodermal cells, the upregulation of KDM6a was highest in endodermal cells. The alterations of its expression suggest its role in chromatin remodeling after RV infection described above. This motivated us to estimate the expression patterns of other genes encoding components of the SWI/SNF and of the NURF chromatin remodeling complexes [9] by mapping them into the SOM (Supplement Figure S4B). We found that, indeed, Smarcc2, Smarcd3, and Btpf were all upregulated in endoderm-derived cells after RV infection, which further supports the assumption that SWI/SNF and possibly also NURF contribute to chromatin remodeling during RV infection. In conclusion, expression changes of different sets of genes involved in epigenetic regulation and of constituents of the ATP-dependent chromatin remodeling complexes such as KDM6a-UTX were detected in association with RV-infection, especially during endodermal differentiation.

### 3.5. RV Infection Impairs Aggregation of iPSCs into Embryoid Bodies

**Figure 5:**
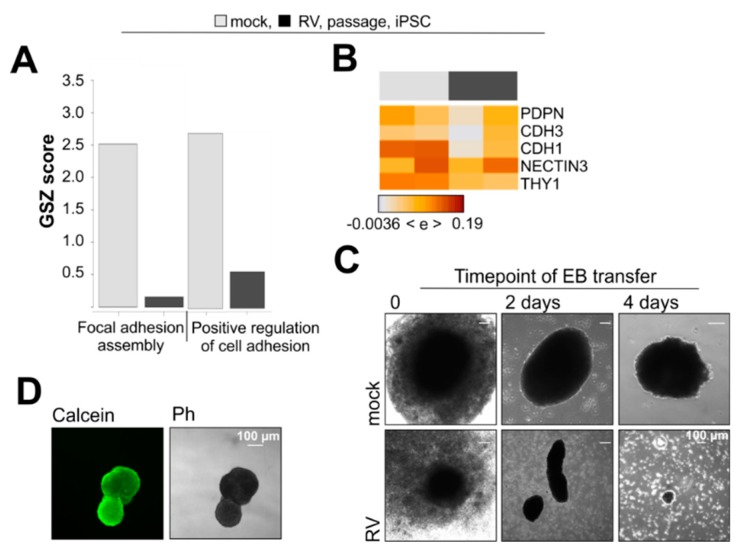
RV impairs the cellular adhesion capacity of iPSCs. (**A**) Gene signatures related to focal adhesion in mock- and RV-infected iPSCs. (**B**) Expression heatmap of selected marker genes involved in cellular adhesion. (**C**) Assessment of 3D stability through embryoid body (EB) formation. Shown are images before and after cultivation in suspension. (**D**) To verify viability, EBs were stained with calcein after cultivation for two weeks.

The progression of embryogenesis does not only involve the activation of developmental pathways, but also requires cell–cell interactions based on adhesive forces [35]. The relevance of these observations for RV-infected iPSCs was emphasized by gene ontology analysis regarding focal adhesion and regulation of cell adhesion (Figure 5A). Transcriptomic analysis revealed that THY-1 (also known as cluster of differentiation (CD) 90) was among the targets affected by RV (Figure 5B). The relevance of THY-1 (CD90) for cellular adhesion capacity was highlighted for CD90 negative carcinoma, which compared to their CD90 positive counterparts lack the ability to form spheres [36]. Accordingly, we have addressed whether RV alters the spontaneous aggregation capacity of iPSCs into 3D aggregates called embryoid bodies (EBs). EB formation relies on cell–cell adhesive interactions [35]. Compared to the mock control, EBs generated from RV-infected iPSCs were reduced in diameter and of irregular shape (Figure 5C). Furthermore, during cultivation they lost stability and small-sized debris was generated (Figure 5C). In contrast to RV-infected iPSCs, the mock-infected controls generated viable EBs as indicated by staining with calcein performed after two weeks of cultivation (Figure 5D). In conclusion, RV infection impaired the adhesion capacity of iPSCs as shown by their reduced ability to assemble into EBs. This suggests an impaired cell–cell interaction capacity during lineage segregation.

### 3.6. RV Infection Specifically Affects Developmental Pathways during Endodermal Differentiation

**Figure 6:**
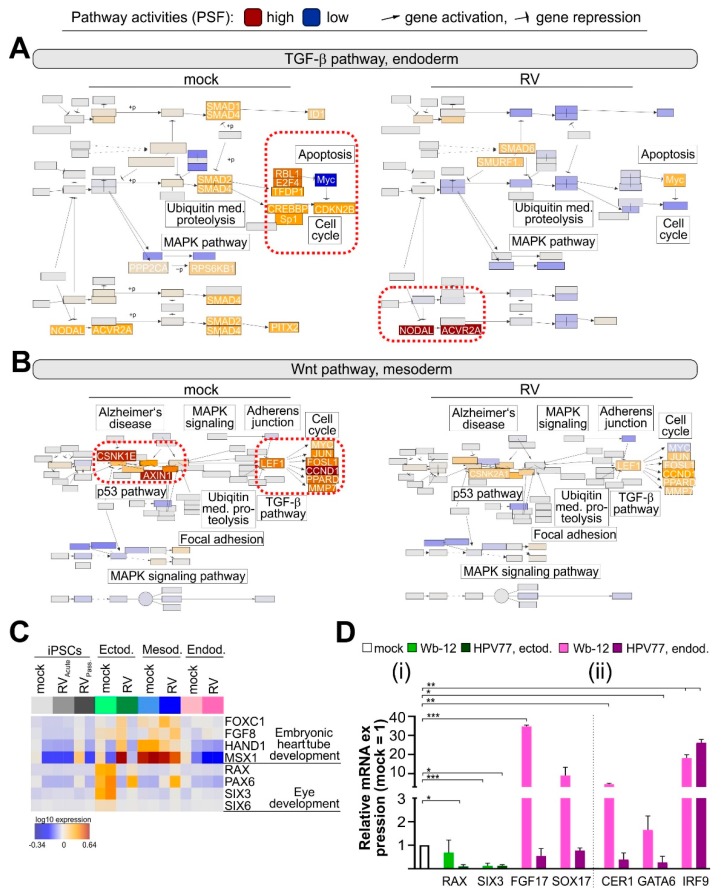
RV exerts lineage-specific effects on developmental signaling pathways and alters expression of transcription and growth factors. Pathway signal flow (PSF) activity plot of (**A**) the TGF-β signaling pathway in endodermal cells (highlighted are genes with higher (NODAL, ACVR2A, Myc) and lower (RBL1 and E2F4) activity after RV infection) and (**B**) of the Wnt signaling pathway in mesodermal cells (highlighted are genes with lower (the CSNK1E/AXIN1E and the LEF1/CCND1 axis) activity after RV infection). The calculation of the activity of the nodes was based on the PSF algorithm using the respective gene expression values and the wirings between the nodes [21]. (**C**) Selected genes within pathways that were specifically affected by RV infection are illustrated in the heatmap of the transcriptome of mock- and RV-infected iPSCs and iPSC-derived lineages. (**D**) For qRT-PCR expression analysis, the 2^−∆∆Ct^ method based on normalization to HPRT1 gene was used. Values are given as means ± SD (n = 4 for Wb-12-infected ectoderm, n = 2 for HPV77-infected ectoderm, otherwise n = 3). See also Figures S5–S7.

For assessment of the effect of RV on global cellular signaling networks, we focused on two important signaling pathways, namely transforming growth factor β (TGF-β) and Wnt/β-catenin (Wnt), (Figure 6A,B, respectively). The TGF-β signaling pathway is involved in cell growth and differentiation during embryogenesis [37]. The Wnt signaling pathway regulates the interaction between cellular pathways involved in primary germ layer formation and is required for mesodermal differentiation from pluripotent stem cells [38]. A more detailed view of the TGF-β and Wnt signaling pathways is provided in Supplementary Figures S5 and S6, respectively. As expected, the highest TGF-β signaling pathway activity was observed in endodermal cells, while the Wnt signaling pathway was most active in mesodermal cells (Supplement Figures S5 and S6). Thus, these two lineages were depicted in Figure 6A,B, respectively, to highlight the effect of RV on their activity in comparison to the respective controls. Within the TGF-β signaling pathway, mock-infected endodermal cells show high cell cycle activity induced by CDKN2B and its downstream interaction partners, which became deactivated during RV infection (Figure 6A and Supplement Figure S5). Additionally, RV infection in endodermal cells was specifically accompanied by a strong activation of NODAL, an essential component of the TGF-β signaling pathway (Figure 6A and Supplement Figure S5). During RV infection, the transcriptional activity of Wnt signaling pathway was reduced in mesodermal cells (Figure 6B), whereas for ectodermal and endodermal cells, almost no alteration in its activity was detected (Supplement Figure S6).

As congenital rubella leads to defects in heart and eye development, we analyzed the impact of RV infection on the underlying molecular pathways. For members of the gene annotation embryonic heart tube development and the gene set heart morphogenesis only a slight effect of RV infection was noted (Supplement Figure S7), suggesting the involvement of other factors. In mesodermal cells, expression of HAND1, which is involved in embryonic heart tube development, was reduced after RV infection as compared to the mock controls (Figure 6C and Figure 1E). The ectoderm gives rise to components of the eye. At the molecular level, RV infection of ectodermal cells impaired the gene set eye development (Figure 6C), (Supplement Figure S7). Specifically, SIX3 and SIX6, as key transcription factors for mammalian eye development [39,40], were reduced in their expression level (Figure 6C). Among others, SIX3, together with RAX, initiates transcription of genes required for lens placode formation [39].

To further assess developmental pathways with relevance for the teratogenic outcome of RV infection, we determined the mRNA expression of RAX and SIX3 (as important factors for eye development) besides FGF17 (Fibroblast Growth Factor 17) and SOX17 (as contributing factors for endodermal differentiation and cardiovascular development) by RT-qPCR in ectodermal and endodermal cells, respectively (Figure 6D). Here, the vaccine strain HPV77 was used in addition to Wb-12 strain. Attenuated vaccine strains such as HPV77 are not teratogenic as revealed after immunization of unknowingly pregnant women [4]. Thus, any alteration at the molecular level that is present during wild-type Wb-12, but not HPV77 infection, emphasizes its possible contribution to congenital rubella. In comparison to the mock control, a similar reduction in the expression of RAX and SIX3 was detected in ectodermal cells after infection with both RV strains. However, a different picture emerged for FGF17 and SOX17. Figure 6D [i] highlights an increase in the expression of FGF17 and SOX17, which was significant for FGF17 compared to the mock control, but only for endodermal cells derived from Wb-12-infected iPSCs, not for endodermal cells derived from HPV77-infected iPSCs. Moreover, the increase in the expression of the definitive endoderm markers CER and GATA6 after infection with Wb-12 as shown in Figure 2E was not detected after infection with HPV77 (Figure 6D [ii]). However, both RV strains induced a significant increase of the IFN-signaling component IRF9 (Figure 6D [ii]). The endoderm plays an essential role in the crosstalk between the lineages and contributes to the epithelial lining of many organs, including the vascular network. Accordingly, the gene set vasculogenesis, but not angiogenesis, was affected by RV infection (Supplement Figure S7). This emphasizes our notion on the correlation between the impact of RV infection on endodermal cells and congenital rubella.

In summary, specific signatures including the TGF-β signaling pathway were affected by RV infection, but in a lineage-specific manner. In ectodermal cells, RV infection significantly reduced expression of SIX3 as key transcription factors for eye field development. Only for the clinical isolate Wb-12, but not for the vaccine strain HPV77, was an impact on the growth factor FGF17 and the endodermal transcription factor SOX17 noted.

**Figure 7:**
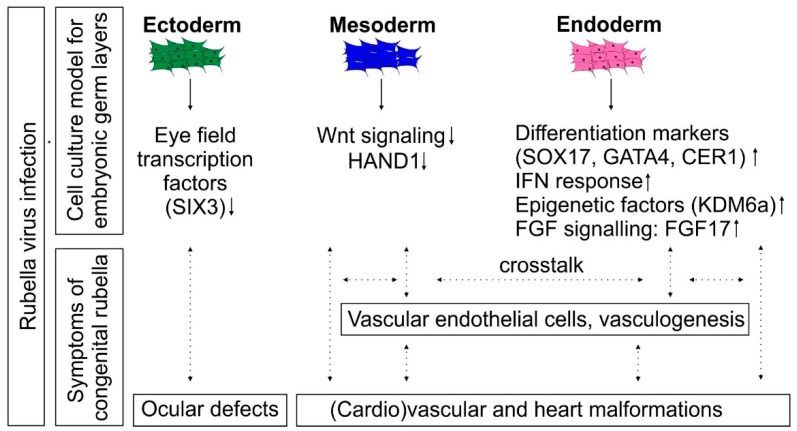
Graphical summary of the identified molecular alterations induced by RV during directed differentiation of iPSCs into the three embryonic germ layer cells. The data was set in a possible relation to the prevailing symptoms of congenital rubella embryopathy. Especially endodermal cells were characterized by profound alterations in their gene expression landscape, including the expression of markers for definitive endoderm and epigenetic factors. This could impair the crosstalk between endodermal and mesodermal cells during differentiation.

Figure 7 summarizes the findings of this study in correlation to the main CRS symptoms. The noncytolytic course of infection of RV during directed differentiation is in agreement with its persistence in multiple organs and tissues during congenital rubella. We have not identified any indication at the molecular level that could contribute to the defects in ear development during congenital rubella. However, sensorineural deafness is often a late-onset symptom and could be associated with pathological alterations in the brain of the infected infants [3].

## 4. Discussion

Knowledge on developmental signaling networks is an essential prerequisite to understand congenital abnormalities, either caused by pathogenic, hereditary or environmental risk factors. Models for developmental toxicity testing range from iPSCs to iPSC-derived EBs and three-dimensional organoids. They have different properties regarding high-throughput screening capacity and relevance for in vivo developmental processes [41]. Their proper assessment requires compounds or pathogens with well-known symptoms arising from embryotoxic or teratogenic alterations during embryonal development. Here, we used RV to correlate clinical observations for congenital rubella syndrome with its impact on the differentiation capacity of iPSCs. Although iPSC-based cell culture models reflect only transient stages during human embryogenesis, they allow us to recapitulate essential developmental pathways that are otherwise inaccessible [42].

Among human pathogens, RV is rather exceptional in its ability to replicate noncytopathically in iPSCs, which in general represent a rather restrictive environment to most viral infections [43]. The protection of human development from a pathogenic insult involves several mechanisms, including transcriptional silencing of viruses in pluripotent stem cells [44] and an intrinsic high expression level of IFN-induced genes [14]. This includes interferon-induced transmembrane protein 1 (IFITM1) and its capacity to restrict the potentially harmful reactivation of human endogenous retroviruses [43]. Otherwise, the antiviral innate immune response in iPSCs is rather refractive [15]. The constitutive overexpression of an active IRF7 as a master regulator of the type I IFN system revealed the harmful effects an activated type I IFN response would have on the expression of pluripotency and lineage specific genes, especially of endodermal cells [45]. In contrast to the engineered type I IFN response in iPSCs through overexpression of IRF7, no morphological changes were noted after infection of iPSCs with RV [13]. However, in agreement with the study on the effect of type I IFNS on differentiation capacity of iPSCs [45], the impact of RV on directed differentiation was most profound during endodermal differentiation. The differences in the signaling cascades of type I and III IFNs [46] might explain the milder effects noted after RV-associated type III IFN activation as compared to the severe effects of an engineered type I IFN response [45]. Our data complements a recent study on the impact of Influenza A virus (IAV) on the pluripotency and proteome of hiPSCs [47]. Whereas, in contrast to RV, IAV reduces the pluripotency of iPSCs, both virus infections induce ISG15 and IFN λ1 [47], highlighting this observation as an innate immune mechanism that is already developed in iPSCs. Further studies need to address whether the impact of RV infection on endodermal differentiation is correlated with the activation of the type III IFN signaling pathway and how this affects the course of infection of RV in iPSCs. In ectodermal cells, RV infection was associated with the downregulation of SIX3, an essential transcription factor for early eye development [48]. Together with SIX6, SIX3 suppresses Wnt signaling, which could contribute to the slight activation of this essential developmental signaling pathway in ectodermal cells derived from RV-infected iPSCs [40]. Their functional importance during retinal development and eye field specification was recently shown by the use of iPSC-derived retinal organoids [39]. Our study complements a previous study on the gene expression profile of fetal (HUVEC originating from umbilical cord veins) and adult (HSaVEC derived from the saphenous vein) endothelial cells which revealed a specific enrichment of 18 downregulated genes within the GO terms “sensory organ development”, “eye development”, and “ear development” [49].

Among the embryonic germ layers, especially differentiation to definite endoderm appeared to be affected by RV infection. In addition to its role in formation of organs of the digestive tract, the interaction of endodermal cells with precardiac mesoderm drives specification and differentiation of cardiac myocytes and cells of endocardial endothelium [50]. This is supported by studies on the contribution of signals from endodermal cells and the interactive crosstalk between the endoderm and mesoderm to differentiation of ESCs to a cardiomyogenic lineage [9]. RV infection does not only target the endoderm, but also signals that facilitate this interactive crosstalk. This includes Cerberus as a bone morphogenetic protein (BMP) antagonist [51]. The secretion of Cerberus from endodermal cells initiates differentiation of the neighboring tissue, namely the overlying cardiac mesoderm [51,52]. Furthermore, the analysis of endoderm-depleted frog and avian embryos revealed that the endoderm contributes to vasculogenesis and vascular tube formation [53]. Thus, as summarized in Figure 7, the molecular events identified in RV-infected endodermal cells could contribute to cardiovascular defects during congenital rubella [2].

Besides the mere expression level of essential components of developmental pathways, post-translational histone modifications are involved in the regulation of gene expression during development. The balance between H3K4me as an active and H3K27 as an inactive state histone modification directs the switch between active and inactive pathways during differentiation [54]. The activity of the KDM6A (UTX) demethylase was especially upregulated in endodermal cells during RV infection. KDM6A demethylase activity was reported to counteract DNA damage response and cell death induction in differentiating ESCs [55], which could also apply to RV-infected endodermal cells.

RV infection was associated with an upregulation of definitive endoderm-enriched transcription factors, including GATA4, EOMES, and SOX17 [56]. In a context- and dose-dependent manner, the transcription factor EOMES directs cardiac development as well as endoderm specification [57]. Whereas SOX17-null mice revealed a downregulation of several genes involved in heart development [58], the ectopic overexpression of SOX17 during hematopoiesis impaired survival of early hematopoietic precursors due to induction of apoptosis [59]. This indicates that normal embryonal development, especially cardiac specification, requires fine-tuned expression of several factors [60], which appears to be affected by RV infection.

The characterization of teratogens such as RV on iPSC-based models is an essential requirement to emphasize their suitability for the assessment of embryotoxicants and to identify relevant parameters to increase their predictive power. Congenital heart malformations are not only caused by pathogens such as RV, they are the most common among human developmental defects identified for human births. iPSC-based models enable valuable insights into human development and processes that might disturb its normal progression, which will broaden our diagnostic and treatment options for congenital defects. Further studies are needed to correlate the identified transcriptional changes with functional consequences for pathways directing embryonal development.

## Supplementary Materials

The following are available online at https://www.mdpi.com/2073-4409/8/8/870/s1, Table S1: Related to description of quantitative real-time PCR analysis, Table S2: Related to Figure 2C. Spot cluster characteristics, Figure S1: Mean expression profiles of ‘spot’-clusters of genes which were denoted with capital letters A–E., Figure S2: Gene set enrichment for pathway analysis of mock- and RV-infected iPSCs and iPSC-derived lineages., Figure S3: Characterization of the IFN-response gene signature in RV-infected iPSCs and iPSC-derived lineages., Figure S4: Characterization of gene expression signatures related to epigenetic regulation., Figure S5: Pathway signal flow (PSF) activity plot of the TGF-beta signaling pathway in ecto-, meso-, and endodermal cells derived from mock- and RV-infected iPSCs., Figure S6: Pathway signal flow (PSF) activity plot of the Wnt signaling pathway in ecto-, meso-, and endodermal cells derived from mock- and RV-infected iPSCs., Figure S7: Gene set expression signatures of developmental programs in mock- and RV-infected iPSCs and iPSC-derived lineages.

## References

[1] Dudgeon J.A. (1975). Congenital rubella. J. Pediatr..

[2] Duszak R.S. (2009). Congenital rubella syndrome—Major review. Optometry.

[3] Lazar M., Perelygina L., Martines R., Greer P., Paddock C.D., Peltecu G., Lupulescu E., Icenogle J., Zaki S.R. (2016). Immunolocalization and distribution of rubella antigen in fatal congenital rubella syndrome. EBioMedicine.

[4] Freij B.J., South M.A., Sever J.L. (1988). Maternal rubella and the congenital rubella syndrome. Clin. Perinatol..

[5] Bouthry E., Picone O., Hamdi G., Grangeot-Keros L., Ayoubi J.M., Vauloup-Fellous C. (2014). Rubella and pregnancy: Diagnosis, management and outcomes. Prenat. Diagn..

[6] Enders G., Nickerl-Pacher U., Miller E., Cradock-Watson J.E. (1988). Outcome of confirmed periconceptional maternal rubella. Lancet.

[7] Rossant J., Tam P.P.L. (2018). Exploring early human embryo development. Science.

[8] Kugler J., Huhse B., Tralau T., Luch A. (2017). Embryonic stem cells and the next generation of developmental toxicity testing. Expert Opin. Drug Metab. Toxicol..

[9] Van Vliet P., Wu S.M., Zaffran S., Puceat M. (2012). Early cardiac development: A view from stem cells to embryos. Cardiovasc. Res..

[10] Simunovic M., Brivanlou A.H. (2017). Embryoids, organoids and gastruloids: New approaches to understanding embryogenesis. Development.

[11] Meganathan K., Jagtap S., Wagh V., Winkler J., Gaspar J.A., Hildebrand D., Trusch M., Lehmann K., Hescheler J., Schluter H. (2012). Identification of thalidomide-specific transcriptomics and proteomics signatures during differentiation of human embryonic stem cells. PLoS ONE.

[12] Luz A.L., Tokar E.J. (2018). Pluripotent stem cells in developmental toxicity testing: A review of methodological advances. Toxicol. Sci..

[13] Hubner D., Jahn K., Pinkert S., Bohnke J., Jung M., Fechner H., Rujescu D., Liebert U.G., Claus C. (2017). Infection of ipsc lines with miscarriage-associated coxsackievirus and measles virus and teratogenic rubella virus as a model for viral impairment of early human embryogenesis. ACS Infect. Dis..

[14] Wu X., Dao Thi V.L., Huang Y., Billerbeck E., Saha D., Hoffmann H.H., Wang Y., Silva L.A.V., Sarbanes S., Sun T. (2018). Intrinsic immunity shapes viral resistance of stem cells. Cell.

[15] Hong X.X., Carmichael G.G. (2013). Innate immunity in pluripotent human cells: Attenuated response to interferon-beta. J. Biol. Chem..

[16] Dziedzicka D., Markouli C., Barbe L., Spits C., Sermon K., Geens M. (2016). A high proliferation rate is critical for reproducible and standardized embryoid body formation from laminin-521-based human pluripotent stem cell cultures. Stem Cell Rev..

[17] Wirth H., Loffler M., von Bergen M., Binder H. (2011). Expression cartography of human tissues using self organizing maps. BMC Bioinform..

[18] Subramanian A., Tamayo P., Mootha V.K., Mukherjee S., Ebert B.L., Gillette M.A., Paulovich A., Pomeroy S.L., Golub T.R., Lander E.S. (2005). Gene set enrichment analysis: A knowledge-based approach for interpreting genome-wide expression profiles. Proc. Natl. Acad. Sci. USA.

[19] Wirth H., von Bergen M., Murugaiyan J., Rosler U., Stokowy T., Binder H. (2012). Maldi-typing of infectious algae of the genus prototheca using som portraits. J. Microbiol. Methods.

[20] Loffler-Wirth H., Kalcher M., Binder H. (2015). Opossom: R-package for high-dimensional portraying of genome-wide expression landscapes on bioconductor. Bioinformatics.

[21] Nersisyan L., Löffler-Wirth H., Arakelyan A., Binder H. (2014). Gene set- and pathway- centered knowledge discovery assigns transcriptional activation patterns in brain, blood, and colon cancer: A bioinformatics perspective. Int. J. Knowl. Discov. Bioinform..

[22] Claus C., Bergs S., Emmrich N.C., Hubschen J.M., Mankertz A., Liebert U.G. (2017). A sensitive one-step taqman amplification approach for detection of rubella virus clade i and ii genotypes in clinical samples. Arch. Virol..

[23] Hellemans J., Mortier G., De Paepe A., Speleman F., Vandesompele J. (2007). Qbase relative quantification framework and software for management and automated analysis of real-time quantitative pcr data. Genome Biol..

[24] Wang H., Zhang K., Liu Y., Fu Y., Gao S., Gong P., Wang H., Zhou Z., Zeng M., Wu Z. (2017). Telomere heterogeneity linked to metabolism and pluripotency state revealed by simultaneous analysis of telomere length and rna-seq in the same human embryonic stem cell. BMC Biol..

[25] Ben-Porath I., Thomson M.W., Carey V.J., Ge R., Bell G.W., Regev A., Weinberg R.A. (2008). An embryonic stem cell-like gene expression signature in poorly differentiated aggressive human tumors. Nat. Genet..

[26] Chu L.F., Leng N., Zhang J., Hou Z., Mamott D., Vereide D.T., Choi J., Kendziorski C., Stewart R., Thomson J.A. (2016). Single-cell rna-seq reveals novel regulators of human embryonic stem cell differentiation to definitive endoderm. Genome Biol..

[27] Hopp L., Loeffler-Wirth H., Nersisyan L., Arakelyan A., Binder H. (2018). Footprints of sepsis framed within community acquired pneumonia in the blood transcriptome. Front Immunol.

[28] Andres-Terre M., McGuire H.M., Pouliot Y., Bongen E., Sweeney T.E., Tato C.M., Khatri P. (2015). Integrated, multi-cohort analysis identifies conserved transcriptional signatures across multiple respiratory viruses. Immunity.

[29] Sweeney T.E., Wong H.R., Khatri P. (2016). Robust classification of bacterial and viral infections via integrated host gene expression diagnostics. Sci. Transl. Med..

[30] Schmidtke L., Schrick K., Saurin S., Kafer R., Gather F., Weinmann-Menke J., Kleinert H., Pautz A. (2019). The kh-type splicing regulatory protein (ksrp) regulates type iii interferon expression post-transcriptionally. Biochem. J..

[31] Winzen R., Thakur B.K., Dittrich-Breiholz O., Shah M., Redich N., Dhamija S., Kracht M., Holtmann H. (2007). Functional analysis of ksrp interaction with the au-rich element of interleukin-8 and identification of inflammatory mrna targets. Mol. Cell. Biol..

[32] Dambacher S., Hahn M., Schotta G. (2010). Epigenetic regulation of development by histone lysine methylation. Heredity.

[33] Thalheim T., Hopp L., Binder H., Aust G., Galle J. (2018). On the cooperation between epigenetics and transcription factor networks in the specification of tissue stem cells. Epigenomes.

[34] Grandy R.A., Whitfield T.W., Wu H., Fitzgerald M.P., VanOudenhove J.J., Zaidi S.K., Montecino M.A., Lian J.B., van Wijnen A.J., Stein J.L. (2016). Genome-wide studies reveal that h3k4me3 modification in bivalent genes is dynamically regulated during the pluripotent cell cycle and stabilized upon differentiation. Mol. Cell. Biol..

[35] Bratt-Leal A.M., Carpenedo R.L., McDevitt T.C. (2009). Engineering the embryoid body microenvironment to direct embryonic stem cell differentiation. Biotechnol. Prog..

[36] Zhang K.T., Che S.Y., Su Z., Zheng S.Y., Zhang H.Y., Yang S.L., Li W.D., Liu J.P. (2018). Cd90 promotes cell migration, viability and sphere-forming ability of hepatocellular carcinoma cells. Int. J. Mol. Med..

[37] Liu C., Peng G., Jing N. (2018). Tgf-beta signaling pathway in early mouse development and embryonic stem cells. Acta Biochim. Biophys. Sin..

[38] Lindsley R.C., Gill J.G., Kyba M., Murphy T.L., Murphy K.M. (2006). Canonical wnt signaling is required for development of embryonic stem cell-derived mesoderm. Development.

[39] Weed L.S., Mills J.A. (2017). Strategies for retinal cell generation from human pluripotent stem cells. Stem Cell Investig..

[40] Diacou R., Zhao Y., Zheng D., Cvekl A., Liu W. (2018). Six3 and six6 are jointly required for the maintenance of multipotent retinal progenitors through both positive and negative regulation. Cell Rep..

[41] Worley K.E., Rico-Varela J., Ho D., Wan L.Q. (2018). Teratogen screening with human pluripotent stem cells. Integr. Biol..

[42] Rathjen J. (2014). The states of pluripotency: Pluripotent lineage development in the embryo and in the dish. ISRN Stem Cells.

[43] Fu Y., Zhou Z., Wang H., Gong P., Guo R., Wang J., Lu X., Qi F., Liu L. (2017). Ifitm1 suppresses expression of human endogenous retroviruses in human embryonic stem cells. FEBS Open Bio.

[44] Wolf D., Goff S.P. (2009). Embryonic stem cells use zfp809 to silence retroviral dnas. Nature.

[45] Eggenberger J., Blanco-Melo D., Panis M., Brennand K.J., tenOever B.R. (2019). Type i interferon response impairs differentiation potential of pluripotent stem cells. Proc. Natl. Acad. Sci. USA.

[46] Pervolaraki K., Talemi S.R., Albrecht D., Bormann F., Bamford C., Mendoza J.L., Garcia K.C., McLauchlan J., Hofer T., Stanifer M.L. (2018). Differential induction of interferon stimulated genes between type i and type iii interferons is independent of interferon receptor abundance. PLoS Pathog..

[47] Zahedi-Amiri A., Sequiera G.L., Dhingra S., Coombs K.M. (2019). Influenza a virus-triggered autophagy decreases the pluripotency of human-induced pluripotent stem cells. Cell Death Dis..

[48] Heavner W., Pevny L. (2012). Eye development and retinogenesis. Cold Spring Harb. Perspect. Biol..

[49] Geyer H., Bauer M., Neumann J., Ludde A., Rennert P., Friedrich N., Claus C., Perelygina L., Mankertz A. (2016). Gene expression profiling of rubella virus infected primary endothelial cells of fetal and adult origin. Virol. J..

[50] Lough J., Sugi Y. (2000). Endoderm and heart development. Dev. Dyn..

[51] Mulloy B., Rider C.C. (2015). The bone morphogenetic proteins and their antagonists. Vitam. Horm..

[52] Foley A.C., Korol O., Timmer A.M., Mercola M. (2007). Multiple functions of cerberus cooperate to induce heart downstream of nodal. Dev. Biol..

[53] Vokes S.A., Krieg P.A. (2002). Endoderm is required for vascular endothelial tube formation, but not for angioblast specification. Development.

[54] Mikkelsen T.S., Ku M., Jaffe D.B., Issac B., Lieberman E., Giannoukos G., Alvarez P., Brockman W., Kim T.K., Koche R.P. (2007). Genome-wide maps of chromatin state in pluripotent and lineage-committed cells. Nature.

[55] Hofstetter C., Kampka J.M., Huppertz S., Weber H., Schlosser A., Muller A.M., Becker M. (2016). Inhibition of kdm6 activity during murine esc differentiation induces DNA damage. J. Cell Sci..

[56] Fisher J.B., Pulakanti K., Rao S., Duncan S.A. (2017). Gata6 is essential for endoderm formation from human pluripotent stem cells. Biol Open.

[57] Pfeiffer M.J., Quaranta R., Piccini I., Fell J., Rao J., Ropke A., Seebohm G., Greber B. (2018). Cardiogenic programming of human pluripotent stem cells by dose-controlled activation of eomes. Nat. Commun..

[58] Pfister S., Jones V.J., Power M., Truisi G.L., Khoo P.L., Steiner K.A., Kanai-Azuma M., Kanai Y., Tam P.P., Loebel D.A. (2011). Sox17-dependent gene expression and early heart and gut development in sox17-deficient mouse embryos. Int. J. Dev. Biol..

[59] Serrano A.G., Gandillet A., Pearson S., Lacaud G., Kouskoff V. (2010). Contrasting effects of sox17- and sox18-sustained expression at the onset of blood specification. Blood.

[60] George R.M., Firulli A.B. (2019). Hand factors in cardiac development. Anat. Rec..

