# Supplementary material for: Teratogenic Rubella Virus Alters the Endodermal Differentiation Capacity of Human Induced Pluripotent Stem Cells
Source: Cells. 2019 Aug 10;8(8):870. doi: 10.3390/cells8080870 (PMC6721684; doi:10.3390/cells8080870)
Supplement: Supplementary file 1 [file cells-08-00870-s001.pdf]

Supplementary Materials:

Teratogenic Rubella Virus Alters the Endodermal Differentiation Capacity of Human Induced Pluripotent Stem Cells

Nicole C. Bilz [1,†], Edith Willscher [2,†], Hans Binder [2], Janik Böhnke [3,‡], Megan L. Stanifer [4], Denise Hübner [1], Steeve Boulant [5,6], Uwe G. Liebert [1] and Claudia Claus [1,*]

[1] Institute of Virology, University of Leipzig, 04103 Leipzig, Germany
[2] Interdisciplinary Center for Bioinformatics, University of Leipzig, 04107 Leipzig, Germany
[3] Institute of Virology, University of Leipzig, 04103 Leipzig, Germany
[4] Schaller Research Group at CellNetworks, Department of Infectious Diseases, Virology, Heidelberg University Hospital, 69120 Heidelberg, Germany
[5] Schaller Research Group at CellNetworks, Department of Infectious Diseases, Virology, Heidelberg University Hospital, 69120 Heidelberg, Germany
[6] Research Group "Cellular Polarity and Viral Infection" (F140), German Cancer Research Center (DKFZ), 69120 Heidelberg, Germany
* Correspondence: claudia.claus@medizin.uni-leipzig.de; Tel.: +49-341-9714321
† These authors contributed equally.
‡ Current address: Institute for Biomedical Engineering, Department of Cell Biology, RWTH Aachen University, Medical School, 52074 Aachen, Germany.

Table S1. Related to description of quantitative real-time PCR analysis. Sequences and respective annealing temperatures (temp) of the primers used in this study.

Gene	Forward primer	Reverse primer	Temp.	References
C1orf43	ACGCCTTTCAAGGGTGTACG	CAAAGACCCCTGTCCCATAGC	58 °C	[1]
HPRT1	TGACACTGGCAAAACAATGCA	GGTCCTTTTCACCAGCAAGCT	62 °C	[2]
Oct4	GAGAACCGAGTGAGAGGCAACC	CATAGTCGCTGCTTGATCGCTTG	58 °C	[3]
PAX6	ACCAGCCCTTCGGTGAAT	TCACTTCCGGGAACTTGAAC	53 °C	[4]
HAND1	CCAAGGATGCACAGTCTGG	CGGTGCGTCCTTTAATCCT	53 °C	[4]
NODAL	GGCAGAAGATGTGGCAGTGG	CAAGTGATGTCGACGGTGC	58 °C	[5]
CXCR4	ACTACACCGAGGAAATGGGCT	CCCACAATGCCAGTTAAGAAGA	56 °C	[6]
MIXL1	CCGAGTCCAGGATCCAGGTA	CTCTGACGCCGAGACTTGG	58 °C	[7]
SOX17	AGATGCTGGGCAAGTCGT	GCTTCAGCCGCTTCACC	54 °C	[7]
FoxA2	AAAAGCCTCCGGTTTCCACTA	TCAGAATCTGCAGGTGCTTGA	56 °C	[8]
EOMES	CACATTGTAGTGGGCAGTGG	CGCCACCAAACTGAGATGAT	58 °C	[7]
GATA4	GAGATGCGTCCCATCAAGAC	GGGAGACGCATAGCCTTGT	56 °C	[9]
GATA6	GCAAAAATACTTCCCCCACA	GCACGGAGGACGTGACTT	53 °C	[10]
CER1	ACTCCGGCTTCTCAGGGGGTC	TGGGTTATAGTCTGGCTGAAGGGCA	64 °C	[7]
IFN λ1	GCAGGTTCAAATCTCTGTCACC	AAGACAGGAGAGCTGCAACTC	60 °C	[11]
IFN λ2/3	GCCACATAGCCCAGTTCAAG	TGGGAGAGGATATGGTGCAG	60 °C	[12]
IRF9	GAGCAGTCCATTCAGACATTGGG	GGCCTCAGTTGTGTCTGTAACTTC	62 °C	[13]
STAT1	CGGCTGAATTTCGGCACCT	CAGTAACGATGAGAGGACCCT	58 °C	§
IFITM1	CCAAGGTCCACCGTGATTAAC	ACCAGTTCAAGAAGAGGGTGTT	56 °C	[6]
IFITM3	GATGTGGATCACGGTGGAC	AGATGCTCAAGGAGGAGCAC	55 °C	[14]
IFIT1	AAAAGCCCACATTTGAGGTG	GAAATTCCTGAAACCGACCA	60 °C	[12]
ISG15	CTGTTCTGGCTGACCTTCG	GGCTTGAGGCCGTACTCC	56 °C	[6]
RAX	GGCCATCCTGGGGTTTACC	GGTCGAGGGGCTTCGTACT	60 °C	[15]
SIX3	ACTACCAGGAGGCCGAGAAG	CAGTTCGCGTTTCTTGCTG	58 °C	[16]
FGF17	TGCTGCCCAACCTCACTC	TCTTTGCTCTTCCCGCTG	54 °C	§
RV genome copies (viral gene p90)				
RV_235	CTG CAC GAG ATY CAG GCC AA CT		60 °C	[17]
RV_419		ACG CAG ATC ACC TCC GCG GT		
TaqMan fluorogenic probe				
RV_291TaqFAM	6FAM-TCA AGA ACG CCG CCA CCT ACG AGC-BBQ			

§ derived from public database RTPrimerDB

Table S2. Related to Figure 2C. Spot cluster characteristics

**Spot # genes (Short name)**	**Upregulated in…**	**Enriched gene sets** [a]	**Top genes** [b]
**A** 251 (Stroma)	Undirected differentiation	Pasini_SUZ12-targets_DN (-56), Konuma_Targets-ofSmad2&3 (-44), HM_ epithelial-mesyenchymal-transition (-38), KEGG_ focal-adhesion (-20)	SPP1, FLNC, ACTG2, ACTC1, NPPB, ANXA2, NTS, CALD1, ADAM19, CDKNA1
**B** 370 (RV-specific)	RV-infected endoderm-	Chaussabel_Interferon-inducable (-13), Hecker_IFN1-targets (-11), HM_IF-Gamma-inducable (-8), Burham_Viral_UP ()-8), BP_Nanog_targets (-7)	CER1, ISG15, CCKBR, RHOBTB, P3H2, SERPINB, SOX17, APOC1, NODAL, CD48, EPSTI1
**C** 1621 (Stemness)	iPSC, endoderm	BP_ESC (<-99), RNA-binding (-87), HM-MYC-targets (58),	IFIT1, IFIT2, LEFTY1, HERC5, MIX1, GAL, PMAIP1, PODXL, POU5F1B, STAT1…
**D** 650 (Ectoderm)	Ectoderm	Wong_embryonic-stem-cell (-12), Nyutten_EZH”-targets_DN (-11), Tirosh-G2M-Phase-genes (-10), BP_cycling-genes (-10),	DLK1, PAMR1, NNAT, SOX21, LHX2, PAX6, SIX3, NOS2, HMGB2, FRZB, RPL14
**E** 583 (Mesoderm)	Mesoderm	GO_Extracellular_exosome (-18), Lee_neural-crest-stem-cell_UP (-11),	DKK1, MSX1, FGF17, GAD1, NKD1, HOXB, RPB1, GPC3, LEF1, COLEC1, SAT1, DLL3
**B’** (IFN-related [b])	RV-infected iPSCs and lineages	Sweeney_Viral_up, Hopp_CAP-viral_UP, Reactome_IFN_a/b_UP	LY6E, IFI27, ISG15, JUP, STAT1, IFI6, IFI7, OAS1, OAS2

[a] Gene sets were taken from published data [18–23] and enrichment p-value (Fishers exact test) decadic exponent is given in brackets; [b] Overlap genes in the IFN-related viral_infection gene sets.

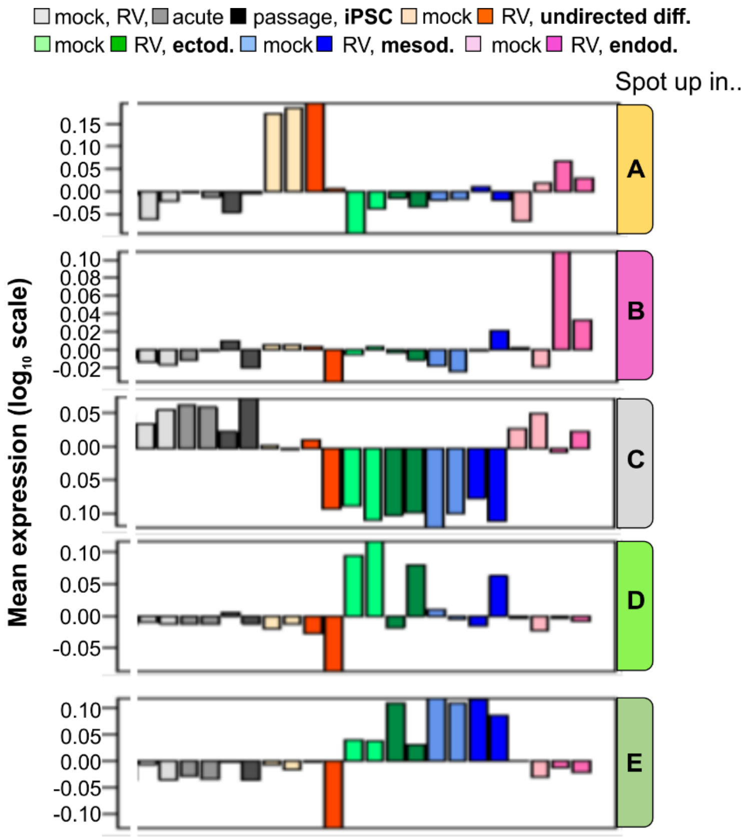

Supplement Figure S1. Mean expression profiles of 'spot'-clusters of genes which were denoted with capital letters A–E. The profiles reveal specific up-regulation of the different cell systems as shown in the legend above.

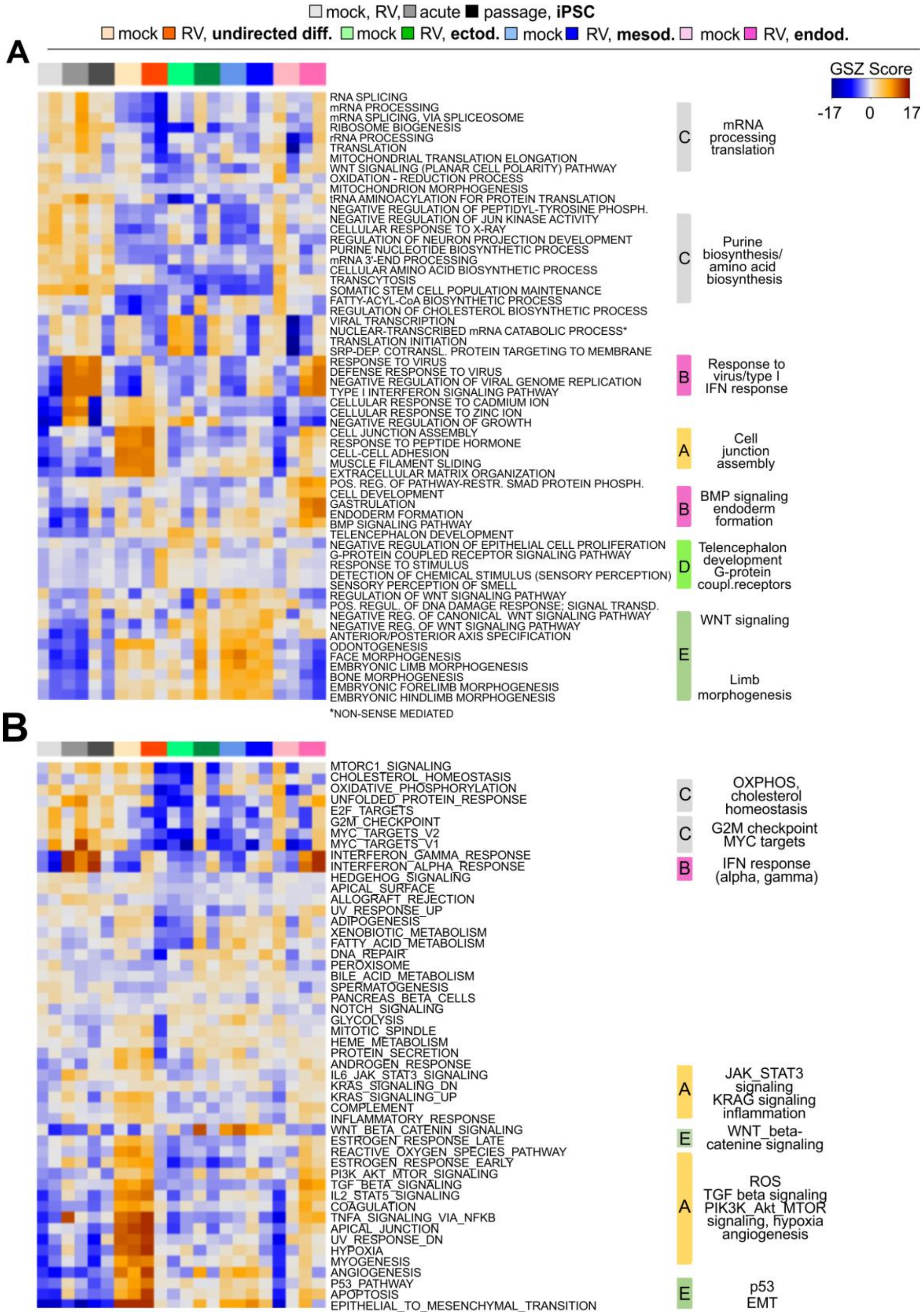

Supplement Figure S2. Gene set enrichment for pathway analysis of mock- and RV-infected iPSCs and iPSC-derived lineages. **(A)** Gene expression heatmap of gene sets of the gene ontology (GO) category ‘biological process‘ (BP), [24]. Hierarchical clustering assigns part of the clusters to the spot clusters obtained by means of SOM-clustering as indicated by the letters on the right together with characteristic functions. **(B)** Gene expression heatmap of gene sets of the category ‘hallmarks of cancer’ (HM) which are of general importance for judging deregulated cell functions also in other, non-cancer diseases [25]. The stemness spot C also accumulates genes reflecting upregulated energy metabolism (oxphos, oxidative) and cell cycle activity while spot A associates with a series of inflammatory and immune response pathways.

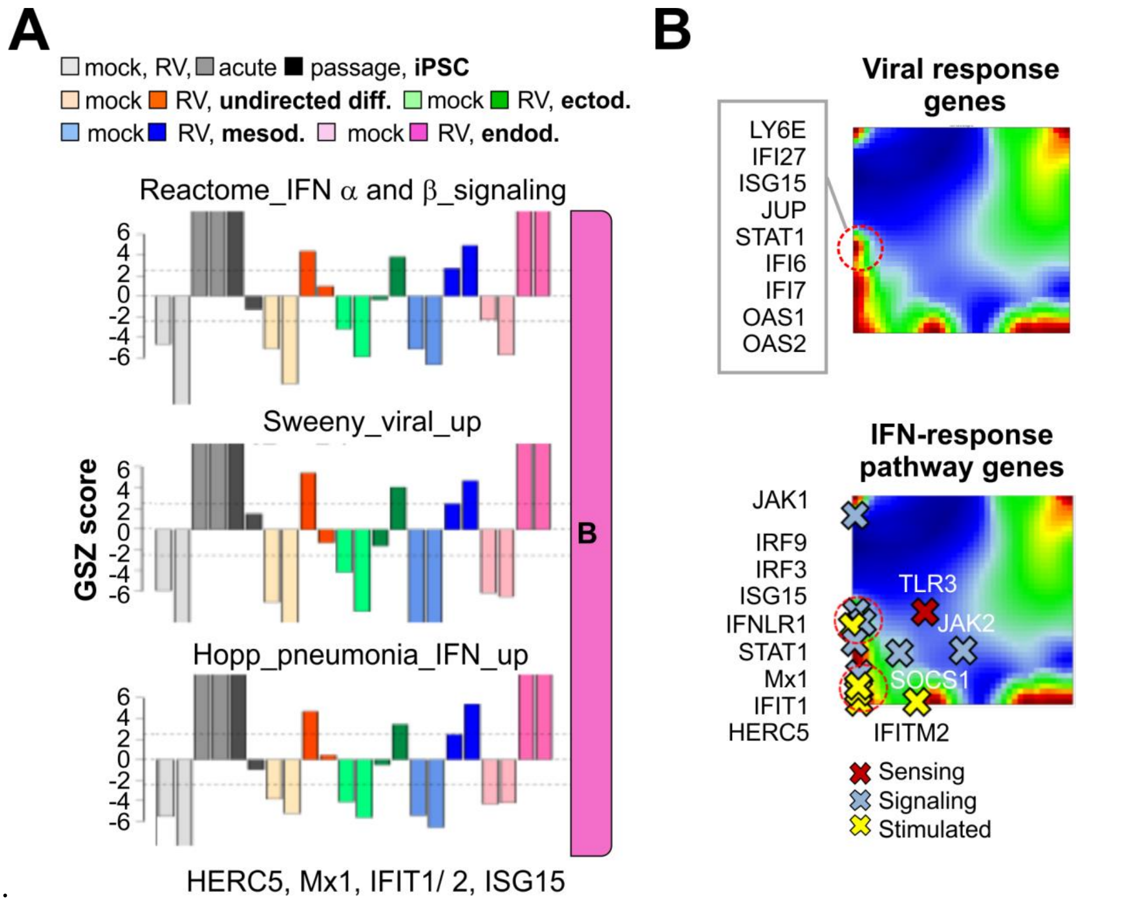

Supplement Figure S3. Characterization of the IFN-response gene signature in RV-infected iPSCs and iPSC-derived lineages. **(A)** Gene set expression signatures reveal activation of IFN-response-related transcriptional programs in RV-infected iPSCs and derived lineages, especially in endodermal cells. **(B)** IFN-response genes with signaling and stimulated functions in the IFN-response pathways accumulate in spots B and C upregulated predominantly in iPS and endoderm-derived cells after RV infection as indicated also by the IFN- and viral response gene signature profiles in part A. They show strong effect of RV in iPS and endodermal cells but only moderate effect in ectoderm- and mesoderm-derived cells. Overlap genes of the viral-response signatures locate in spots B and C and partly overlap also with IFN-pathway genes, selected in part A.

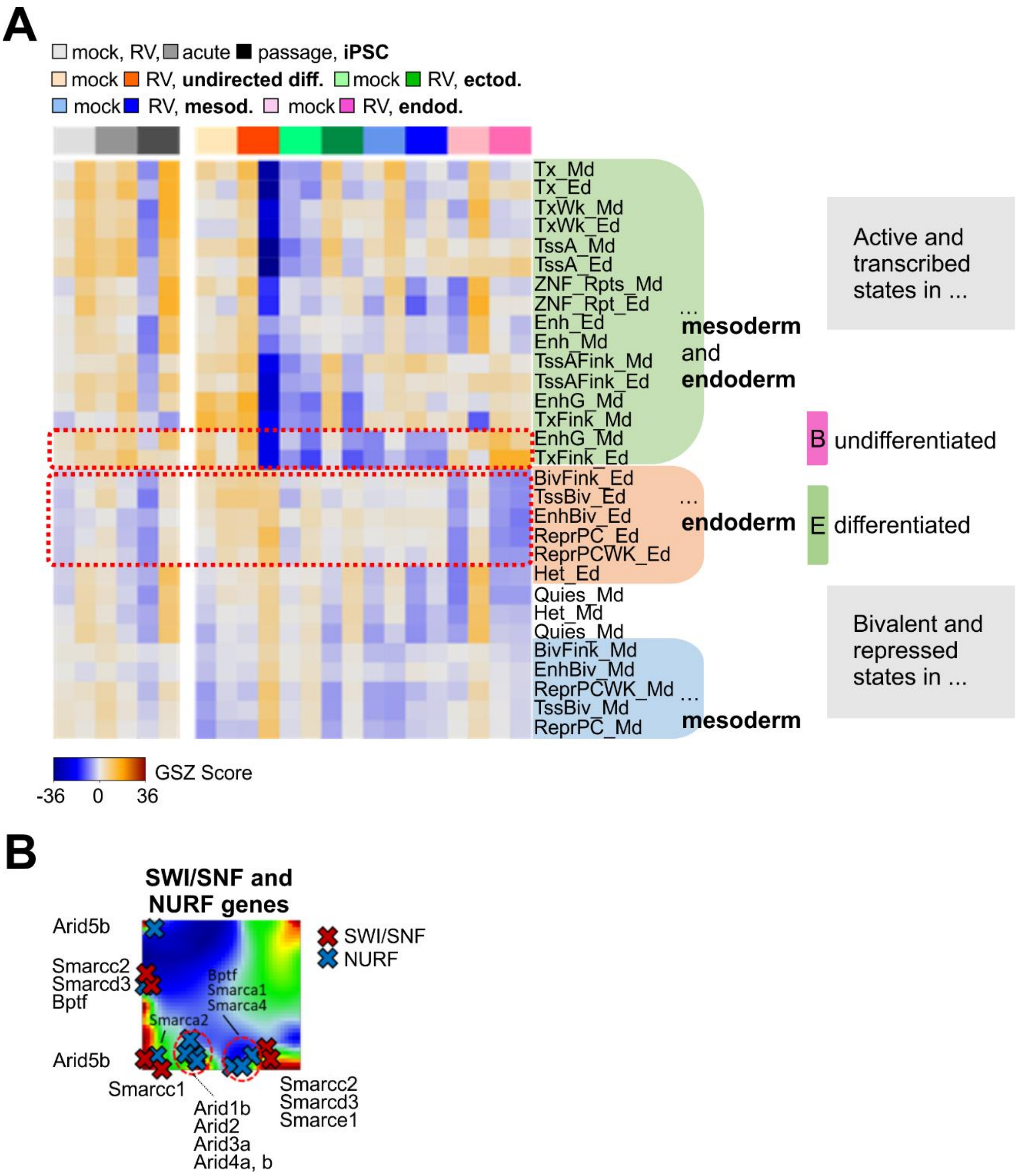

Supplement Figure S4. Characterization of gene expression signatures related to epigenetic regulation. **(A)** Gene expression profiles related to epigenetic regulation, namely of 15 different chromatin states of ES derived mesoderm, ectoderm and endoderm progenitors (www.roadmapepigenomics.org) and of selected chromatin modifying enzymes such as methyltransferases and demethylases of arginine (R) and lysine (K) histone (histone subunit 3, H3) side chains (e.g., H3K4, H3K9, H3K27). TssA are active promoters and Tx transcribed genes including associated enhancer (Enh) and flanking (Flnk) states of the endoderm and mesoderm progenitors cluster together with profiles resembling those of transcription factors regulating organ differentiation (shaded in green). TssBiv indicates genes with bivalent and ReprPC repressed promoters, which form a second cluster (shaded in apricot). **(B)** Expression map with component genes of ATP-dependent remodeling complexes SWI/SNF and NURF.

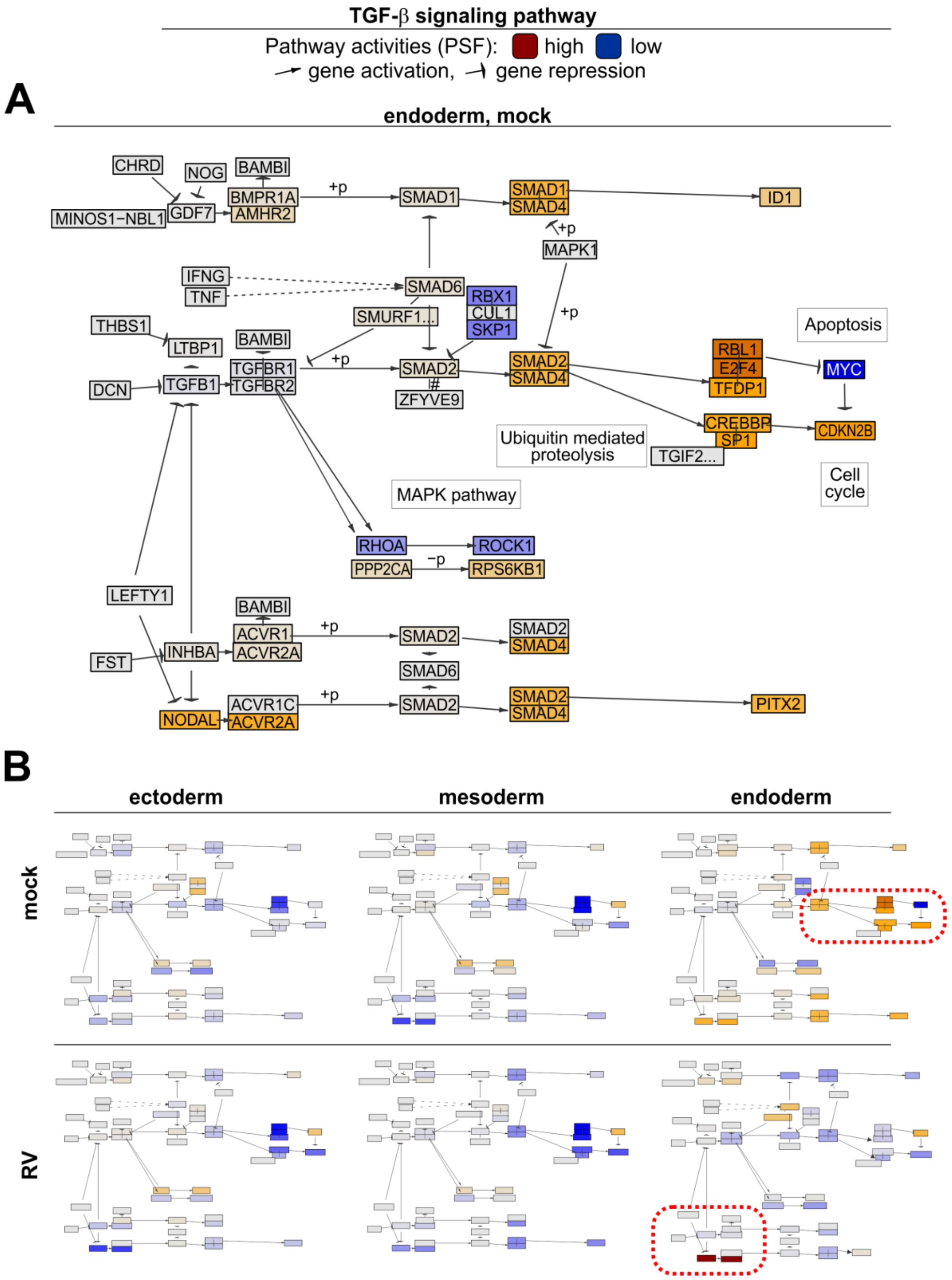

Supplement Figure S5. Pathway signal flow (PSF) activity plot of the TGF-beta signaling pathway in ecto-, meso- and endodermal cells derived from mock- and RV-infected iPSCs. The calculation of the activity of the nodes was based on the PSF-algorithm using the respective gene expression values and the wirings between the nodes [26]. The pathway graphs are shown in the upper part of the figure. The nodes assign the genes which are linked either via activating (arrow-links) or repressing (T-links) interactions according to the respective pathway topology. The dashed areas indicate pathway branches which get specifically upregulated in endoderm-derived cells. Note activation of NODAL and ACVR2A in RV-infected endoderm cells. The lower part of the figure shows the respective pathways with colored nodes where maroon and blue colors assign high and low activity.

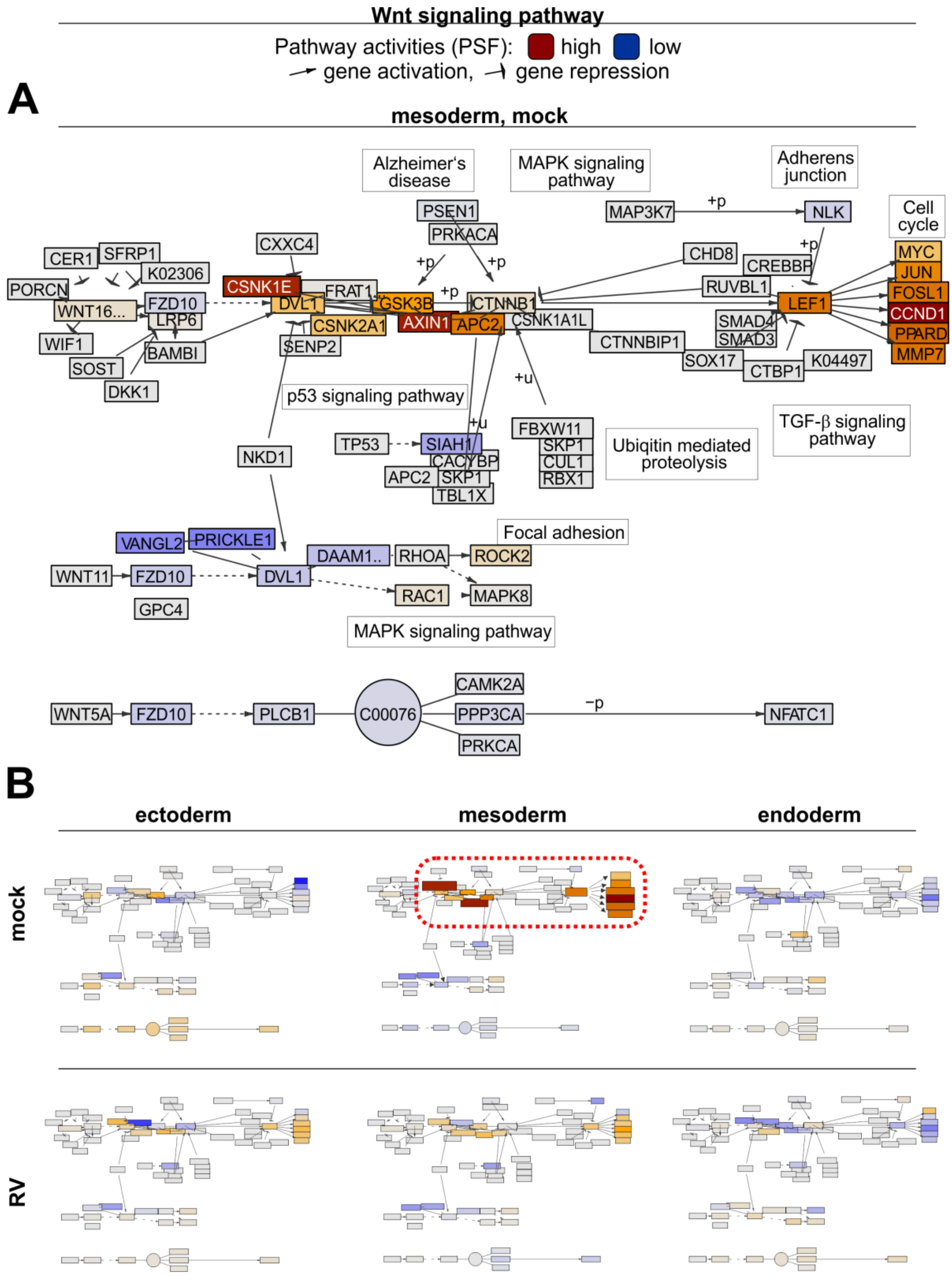

Supplement Figure S6. Pathway signal flow (PSF) activity plot of the Wnt signaling pathway in ecto-, meso- and endodermal cells derived from mock- and RV-infected iPSCs The calculation of the activity of the nodes was based on the PSF-algorithm using the respective gene expression values and the wirings between the nodes [26]. The pathway graphs are shown in the upper part of the figure. The nodes assign the genes which are linked either via activating (arrow-links) or repressing (T-links) interactions according to the respective pathway topology. TGF-beta gets specifically activated in mesoderm-derived cells while it is on low activity levels in endoderm independent of RV-infection. The lower part of the figure shows the respective pathways with colored nodes where maroon and blue colors assign high and low activity.

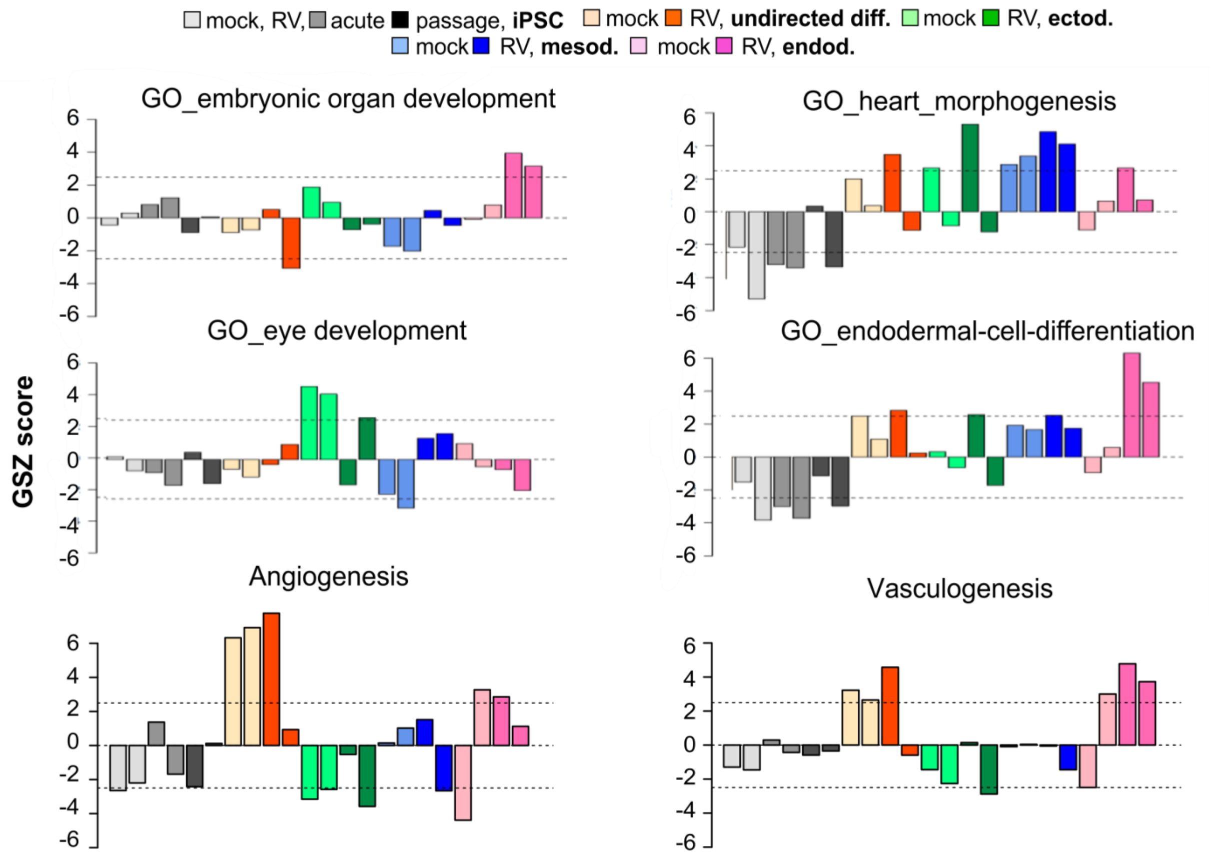

Supplement Figure S7. Gene set expression signatures (Gene Ontology, GO-terms) of developmental programs in mock- and RV-infected iPSCs and iPSC-derived lineages. Highlighted are gene sets involved in organ development, eye development, heart morphogenesis, endodermal cell differentiation, angiogenesis and vasculogenesis.

References

1. Artyukhov, A.S.; Dashinimaev, E.B.; Tsvetkov, V.O.; Bolshakov, A.P.; Konovalova, E.V.; Kolbaev, S.N.; Vorotelyak, E.A.; Vasiliev, A.V. New genes for accurate normalization of qrt-pcr results in study of ips and ips-derived cells. *Gene* **2017**, *626*, 234–240.
2. Cicinnati, V.R.; Shen, Q.; Sotiropoulos, G.C.; Radtke, A.; Gerken, G.; Beckebaum, S. Validation of putative reference genes for gene expression studies in human hepatocellular carcinoma using real-time quantitative rt-pcr. *BMC Cancer* **2008**, *8*, 350.
3. Hirotsu, M.; Setoguchi, T.; Matsunoshita, Y.; Sasaki, H.; Nagao, H.; Gao, H.; Sugimura, K.; Komiya, S. Tumour formation by single fibroblast growth factor receptor 3-positive rhabdomyosarcoma-initiating cells. *Br. J. Cancer* **2009**, *101*, 2030–2037.
4. Pells, S.; Koutsouraki, E.; Morfopoulou, S.; Valencia-Cadavid, S.; Tomlinson, S.R.; Kalathur, R.; Futschik, M.E.; De Sousa, P.A. Novel human embryonic stem cell regulators identified by conserved and distinct cpg island methylation state. *PLoS ONE* **2015**, *10*, e0131102.
5. Dvash, T.; Mayshar, Y.; Darr, H.; McElhaney, M.; Barker, D.; Yanuka, O.; Kotkow, K.J.; Rubin, L.L.; Benvenisty, N.; Eiges, R. Temporal gene expression during differentiation of human embryonic stem cells and embryoid bodies. *Hum. Reprod.* **2004**, *19*, 2875–2883.
6. Wu, X.; Dao Thi, V.L.; Huang, Y.; Billerbeck, E.; Saha, D.; Hoffmann, H.H.; Wang, Y.; Silva, L.A.V.; Sarbanes, S.; Sun, T.; et al. Intrinsic immunity shapes viral resistance of stem cells. *Cell* **2018**, *172*, 423–438 e425.
7. Faial, T.; Bernardo, A.S.; Mendjan, S.; Diamanti, E.; Ortmann, D.; Gentsch, G.E.; Mascetti, V.L.; Trotter, M.W.; Smith, J.C.; Pedersen, R.A. Brachyury and smad signalling collaboratively orchestrate distinct mesoderm and endoderm gene regulatory networks in differentiating human embryonic stem cells. *Development* **2015**, *142*, 2121–2135.
8. Hamasaki, M.; Hashizume, Y.; Yamada, Y.; Katayama, T.; Hohjoh, H.; Fusaki, N.; Nakashima, Y.; Furuya, H.; Haga, N.; Takami, Y.; et al. Pathogenic mutation of alk2 inhibits induced pluripotent stem cell reprogramming and maintenance: Mechanisms of reprogramming and strategy for drug identification. *Stem Cells* **2012**, *30*, 2437–2449.
9. Mills, J.A.; Herrera, P.S.; Kaur, M.; Leo, L.; McEldrew, D.; Tintos-Hernandez, J.A.; Rajagopalan, R.; Gagne, A.; Zhang, Z.; Ortiz-Gonzalez, X.R.; et al. Nipbl(+/-) haploinsufficiency reveals a constellation of transcriptome disruptions in the pluripotent and cardiac states. *Sci. Rep.* **2018**, *8*, 1056.
10. Grandy, R.A.; Whitfield, T.W.; Wu, H.; Fitzgerald, M.P.; VanOudenhove, J.J.; Zaidi, S.K.; Montecino, M.A.; Lian, J.B.; van Wijnen, A.J.; Stein, J.L.; et al. Genome-wide studies reveal that h3k4me3 modification in bivalent genes is dynamically regulated during the pluripotent cell cycle and stabilized upon differentiation. *Mol. Cell. Biol.* **2016**, *36*, 615–627.
11. Bender, S.; Reuter, A.; Eberle, F.; Einhorn, E.; Binder, M.; Bartenschlager, R. Activation of type i and iii interferon response by mitochondrial and peroxisomal mavs and inhibition by hepatitis c virus. *PLoS Pathog.* **2015**, *11*, e1005264.
12. Stanifer, M.L.; Rippert, A.; Kazakov, A.; Willemsen, J.; Bucher, D.; Bender, S.; Bartenschlager, R.; Binder, M.; Boulant, S. Reovirus intermediate subviral particles constitute a strategy to infect intestinal epithelial cells by exploiting tgf-beta dependent pro-survival signaling. *Cell. Microbiol.* **2016**, *18*, 1831–1845.
13. Hong, X.X.; Carmichael, G.G. Innate immunity in pluripotent human cells: Attenuated response to interferon-beta. *J. Biol. Chem.* **2013**, *288*, 16196–16205.
14. Chiang, C.; Beljanski, V.; Yin, K.; Olagnier, D.; Ben Yebdri, F.; Steel, C.; Goulet, M.L.; DeFilippis, V.R.; Streblow, D.N.; Haddad, E.K.; et al. Sequence-specific modifications enhance the broad-spectrum antiviral response activated by rig-i agonists. *J. Virol.* **2015**, *89*, 8011–8025.
15. Sluch, V.M.; Davis, C.H.; Ranganathan, V.; Kerr, J.M.; Krick, K.; Martin, R.; Berlinicke, C.A.; Marsh-Armstrong, N.; Diamond, J.S.; Mao, H.Q.; et al. Differentiation of human escs to retinal ganglion cells using a crispr engineered reporter cell line. *Sci. Rep.* **2015**, *5*, 16595.
16. Zhang, X.; Huang, C.T.; Chen, J.; Pankratz, M.T.; Xi, J.; Li, J.; Yang, Y.; Lavaute, T.M.; Li, X.J.; Ayala, M.; et al. Pax6 is a human neuroectoderm cell fate determinant. *Cell Stem Cell* **2010**, *7*, 90–100.
17. Claus, C.; Bergs, S.; Emmrich, N.C.; Hubschen, J.M.; Mankertz, A.; Liebert, U.G. A sensitive one-step taqman amplification approach for detection of rubella virus clade i and ii genotypes in clinical samples. *Arch. Virol.* **2017**, *162*, 477–486.
18. Pasini, D.; Bracken, A.P.; Hansen, J.B.; Capillo, M.; Helin, K. The polycomb group protein suz12 is required for embryonic stem cell differentiation. *Mol. Cell. Biol.* **2007**, *27*, 3769–3779.

19. Liberzon, A.; Birger, C.; Thorvaldsdottir, H.; Ghandi, M.; Mesirov, J.P.; Tamayo, P. The molecular signatures database hallmark gene set collection. *Cell Syst.* **2015**, *1*, 417–425.
20. Chaussabel, D.; Quinn, C.; Shen, J.; Patel, P.; Glaser, C.; Baldwin, N.; Stichweh, D.; Blankenship, D.; Li, L.; Munagala, I.; et al. A modular analysis framework for blood genomics studies: Application to systemic lupus erythematosus. *Immunity* **2008**, *29*, 150–164.
21. Burnham, K.L.; Davenport, E.E.; Radhakrishnan, J.; Humburg, P.; Gordon, A.C.; Hutton, P.; Svoren-Jabalera, E.; Garrard, C.; Hill, A.V.S.; Hinds, C.J.; et al. Shared and distinct aspects of the sepsis transcriptomic response to fecal peritonitis and pneumonia. *Am. J. Resp. Crit. Care Med.* **2017**, *196*, 328–339.
22. Ben-Porath, I.; Thomson, M.W.; Carey, V.J.; Ge, R.; Bell, G.W.; Regev, A.; Weinberg, R.A. An embryonic stem cell-like gene expression signature in poorly differentiated aggressive human tumors. *Nat. Genet.* **2008**, *40*, 499–507.
23. Tirosh, I.; Izar, B.; Prakadan, S.M.; Wadsworth, M.H., 2nd; Treacy, D.; Trombetta, J.J.; Rotem, A.; Rodman, C.; Lian, C.; Murphy, G.; et al. Dissecting the multicellular ecosystem of metastatic melanoma by single-cell rna-seq. *Science* **2016**, *352*, 189–196.
24. Subramanian, A.; Tamayo, P.; Mootha, V.K.; Mukherjee, S.; Ebert, B.L.; Gillette, M.A.; Paulovich, A.; Pomeroy, S.L.; Golub, T.R.; Lander, E.S.; et al. Gene set enrichment analysis: A knowledge-based approach for interpreting genome-wide expression profiles. *Proc. Natl. Acad. Sci. USA* **2005**, *102*, 15545–15550.
25. Liberzon, A.; Birger, C.; Thorvaldsdottir, H.; Ghandi, M.; Mesirov, J.P.; Tamayo, P. The molecular signatures database hallmark gene set collection. *Cell Syst.* **2015**, *1*, 417–4255.
26. Nersisyan, L.; Löffler-Wirth, H.; Arakelyan, A.; Binder, H. Gene set- and pathway- centered knowledge discovery assigns transcriptional activation patterns in brain, blood, and colon cancer: A bioinformatics perspective. *Int. J. Knowl. Discov. Bioinformatics (IJKDB)* **2014**, *4*, 46–69.

© 2019 by the authors. Licensee MDPI, Basel, Switzerland. This article is an open access article distributed under the terms and conditions of the Creative Commons Attribution (CC BY) license (http://creativecommons.org/licenses/by/4.0/).
